# A stearate-rich diet and oleate restriction directly inhibit tumor growth via the unfolded protein response

**DOI:** 10.1038/s12276-024-01356-2

**Published:** 2024-12-02

**Authors:** Jumpei Ogura, Koji Yamanoi, Kentaro Ishida, Eijiro Nakamura, Shinji Ito, Naoki Aoyama, Yuki Nakanishi, Toshi Menju, Kosuke Kawaguchi, Yuko Hosoe, Mana Taki, Ryusuke Murakami, Ken Yamaguchi, Junzo Hamanishi, Masaki Mandai

**Affiliations:** 1 Department of Gynecology and Obstetrics, Kyoto University Graduate School of Medicine, Kyoto, Japan; 2 Department of Urology and Retroperitoneal Surgery, National Cancer Center Hospital, Tokyo, Japan; 3 Medical Research Support Center, Kyoto University Graduate School of Medicine, Kyoto, Japan; 4 Department of Gastroenterology and Hepatology, Kyoto University Graduate School of Medicine, Kyoto, Japan; 5 Department of Thoracic Surgery, Kyoto University Graduate School of Medicine, Kyoto, Japan; 6 Department of Breast Surgery, Kyoto University Graduate School of Medicine, Kyoto, Japan

**Keywords:** Cancer metabolism, Apoptosis, Cancer metabolism

## Abstract

Fatty acids are known to have significant effects on the properties of cancer cells. Therefore, these compounds have been incorporated into therapeutic strategies. However, few studies have examined the effects of individual fatty acids and their interactions in depth. This study analyzed the effects of various fatty acids on cancer cells and revealed that stearic acid, an abundant saturated fatty acid, had a stronger inhibitory effect on cell growth than did palmitic acid, which is also an abundant saturated fatty acid, by inducing DNA damage and apoptosis through the unfolded protein response (UPR) pathway. Intriguingly, the negative effects of stearate were reduced by the presence of oleate, a different type of abundant fatty acid. We combined a stearate-rich diet with the inhibition of stearoyl-CoA desaturase-1 to explore the impact of diet on tumor growth. This intervention significantly reduced tumor growth in both ovarian cancer models and patient-derived xenografts (PDXs), including those with chemotherapy resistance, notably by increasing stearate levels while reducing oleate levels within the tumors. Conversely, the negative effects of a stearate-rich diet were mitigated by an oleate-rich diet. This study revealed that dietary stearate can directly inhibit tumor growth through mechanisms involving DNA damage and apoptosis mediated by the UPR pathway. These results suggest that dietary interventions, which increase stearic acid levels while decreasing oleic acid levels, may be promising therapeutic strategies for cancer treatment. These results could lead to the development of new cancer treatment strategies.

## Stearic Acid Inhibits Cancer Growth Through DNA Damage

Obesity, characterized by excessive body fat, is linked to higher cancer risks. Researchers explored how specific fatty acids impact cancer growth. The study focused on palmitate, stearate, and oleate, using various cancer cell lines and patient derived xenograft. They found that stearate significantly inhibited cancer cell growth more than palmitate. This was a controlled experiment involving human cancer cell lines and mice fed specialized diets. Results showed that stearate induced DNA damage and cancer cell death, while oleate reduced these effects. The researchers concluded that dietary stearate could suppress tumor growth, especially when combined with inhibitors of fatty acid conversion. Future research could explore dietary interventions as potential cancer treatments.

This summary was initially drafted using artificial intelligence, then revised and fact-checked by the author.

## Introduction

A notable correlation exists between various types of cancers and obesity, which is characterized by an excessive accumulation of body fat. Obesity increases the risk of carcinogenesis^1^. The consumption of a high-fat diet (HFD) augments the malignant potential of cancer cells^2^. Therefore, ingesting an excessive amount of dietary fat is implicated in increasing the risk of cancer development, exacerbating the malignancy of cancer in a tumor-bearing state, and potentially effectuating adverse clinical outcomes^3,4^. However, fatty acids are not universally detrimental, and their effects on cancer cells vary depending on the type of fatty acid^5^.

In biological systems, most fatty acids contain 16 or more carbon atoms and are classified as long-chain fatty acids^6,7^. These long-chain fatty acids can be further categorized into saturated fatty acids (SFAs), which possess only single bonds between carbon atoms, and unsaturated fatty acids (UFAs), which are characterized by the presence of double bonds. UFAs may enhance cancer survival, including stemness and ferroptosis resistance, particularly in renal and ovarian cancers^8–10^, suggesting a potential association between UFAs and adverse clinical outcomes. Conversely, SFAs exert cytotoxic effects on normal cells—especially hepatocytes, endothelial cells, adipocytes, and pancreatic β cells—commonly referred to as lipotoxicity^11–13^. The potential antiproliferative effects of SFAs on cancer cells have been documented recently^14–16^. Caloric restriction markedly increases the proportion of SFAs in the fatty acid composition of biological systems, which may in turn promote tumor suppression^17^. Inhibiting the activity of stearoyl-CoA desaturase (SCD), which catalyzes the conversion of SFAs to UFAs, increases SFA levels and subsequently hinders glioblastoma cell proliferation^18^. An SCD inhibition-mediated increase in the SFA/UFA ratio can enhance antitumor effects on ovarian cancer^16^. Nevertheless, comprehensive studies examining the in vivo effects of SFAs and the subtle differences between them are scarce.

Most studies investigating the roles of SFAs use palmitate for analysis. Palmitate, comprising 16 carbon atoms, is the most abundant SFA in biological systems, and stearate, comprising 18 carbon atoms, is also abundant^6^. They are structurally similar, with the two-carbon atom variation in chain length being the only difference. Therefore, the effects of palmitate and stearate on cells are proposed to have little difference. The two SFA types can have different effects on cancer cells^19^, but few studies have comprehensively explored their cellular effects; the effects of stearate on cancer cells are largely unknown. Moreover, the manipulation of the dietary long-chain fatty acid composition and its in vivo effects remain to be evaluated.

In this study, we aimed to investigate the impacts of palmitate, stearate, and oleate on cancer cells and whether dietary changes would have sufficient clinical impacts. To this end, in addition to a usual HFD, a specialized HFD rich in stearate (S-HFD) was employed to study the differential effects of dietary stearate and oleate on cancer in vivo.

## Materials and methods

### Study approval

The Ethics Committee of the Graduate School and Faculty of Medicine at Kyoto University approved this study (reference numbers G531 and G288). We ensured compliance with the principles of the Declaration of Helsinki. The university’s Animal Research Committee approved the animal experiments conducted in this research.

### Cell culture

The Dr. Melinda Hollingshead from the National Institutes of Health provided the human ovarian cancer cell line OVCAR8. Dr. Iwakoshi, Dr. Masashi Kanai, Dr. Shigeo Takaishi, and Dr. Susan K. Murphy donated the human ovarian cancer cell lines ES-2, OVCAR5, SKOV3, and OVCAR3; the human colon cancer cell lines DLD1 and LoVo; and the human epithelial cell line HOSE^20^, respectively. The American Type Culture Collection (Manassas, VA, USA) supplied the human mammary cancer cell lines MCF-7, MDA-MB-453, MDA-MB-231, and Hs578T; the human mammary epithelial cell line MCF10A; the human lung cancer cell lines NCI-H460, NCI-H1299, and NCI-H1650; and the human colon cancer cell lines HCT116, HT29, and Caco2. The human lung cancer cell line A549 was purchased from the RIKEN BRC cell bank (Tsukuba, Japan). All cell lines, except MCF10A, were cultured in RPMI-1640 medium supplemented with 10% heat-inactivated fetal bovine serum (FBS) and penicillin‒streptomycin. MCF10A cells were grown in DMEM/F12 supplemented with 5% FBS, 0.02% epidermal growth factor, 0.05% insulin, 0.5 μg/mL hydrocortisone, and 1% penicillin‒streptomycin.

### Animal models

BALB/cAJcl-nu/nu mice, aged 4 and 6 weeks, were obtained from CLEA Japan (Tokyo, Japan). Five-week-old nonobese diabetic/Shi-scid IL-2RγKO Jic (NOG) mice were acquired from In Vivo Science, Inc. These animals were maintained under specific pathogen-free conditions. Initially, the mice were provided a standard solid diet, Formula F-2, with 12.0% fat by caloric content and unrestricted access to water. As part of the study design, the mice were subsequently assigned to specific dietary regimens.

### Preparation of mouse xenograft models utilizing human ovarian cancer cell lines

The mice were grouped into experimental categories to study the effects of dietary conditions and therapeutic interventions on tumor growth. Xenografts were established using human ovarian cancer cell lines and genetically modified cells, which were treated with either vehicle or CAY10566. Before transplantation, the mice were acclimatized to the following diets for three days: a normal-fat diet (NFD; 12.0% fat; F-2, Oriental Yeast, Tokyo, Japan), a high-fat diet rich in stearate (S-HFD; 60% fat; D12113001, Research Diets, New Brunswick, NJ, USA), or a high-fat diet rich in oleic acid (O-HFD; 56.7% fat; HFD32, CLEA Japan, Tokyo, Japan), and these diets were maintained throughout the study. Tumor growth was regularly monitored by measuring tumor dimensions, and humane treatment protocols, including euthanasia criteria to prevent excessive tumor growth or ulceration, were strictly followed.

### Mouse xenograft models harboring PDXs

Surgical specimens from patients with ovarian cancer were obtained with informed consent at Kyoto University Hospital and used to create primary xenograft tumors in NOD SCID mice using the Matrigel matrix basement membrane for transplantation. Following tumor establishment, the mice were divided into four treatment groups—NFD+vehicle, NFD + CAY10566, S-HFD + CAY10566, and O-HFD + CAY10566—to assess the effects of dietary conditions and CAY10566 on tumor growth. The tumor volume was periodically measured using methods similar to those employed in ovarian cancer cell line xenograft models to evaluate treatment outcomes.

Detailed descriptions of the in vivo and in vitro analyses, mouse work protocols, preparation of reagents and samples, flow cytometry, enzyme-linked immunosorbent assay (ELISA), RNA sequencing, western blotting, immunohistochemistry (IHC), and liquid chromatography‒mass spectrometry are described in the Supplementary Materials and Methods.

The comprehensive list of reagents and antibodies used for western blotting or IHC, including the corresponding dilutions, primers, short hairpin RNA (shRNA) sequences, and software, along with their sources and research resource identifier numbers, are detailed in the Supplementary Data. Additionally, the detailed compositions of the different diets used in this study are listed in Supplementary Table 1.

### Statistical analyses

At least three independent in vitro experiments and a minimum of two cell lines for in vivo experiments were utilized. The mice in the in vivo studies were randomly assigned to experimental groups. Sample sizes were determined to ensure experimental reproducibility, adhering to the principles of replacement, reduction, and refinement in animal ethics. The results are presented as the means ± standard errors of the means (SEMs). The Mann–Whitney U test was used to analyze data from the in vitro proliferation assays, for group comparisons of ELISA results using in vivo samples, and for assessing tumor volume, weight, and IHC outcomes. The Wilcoxon matched-pairs signed rank test was used to analyze data from the IC_50_ analysis, apoptosis assays, and comparisons of tumor volume and weight between shCtr and shSCD tumors in vivo. An unpaired t test was used to analyze the LC‒MS data. All the statistical analyses were conducted using Prism 10.0.2 software, with significance levels set at *p < 0.05, **p < 0.01, and ***p < 0.001, with ‘ns’ indicating not significant.

## Results

### Stearate inhibits the growth of multiple cancer cell lines

**Fig. 1:**
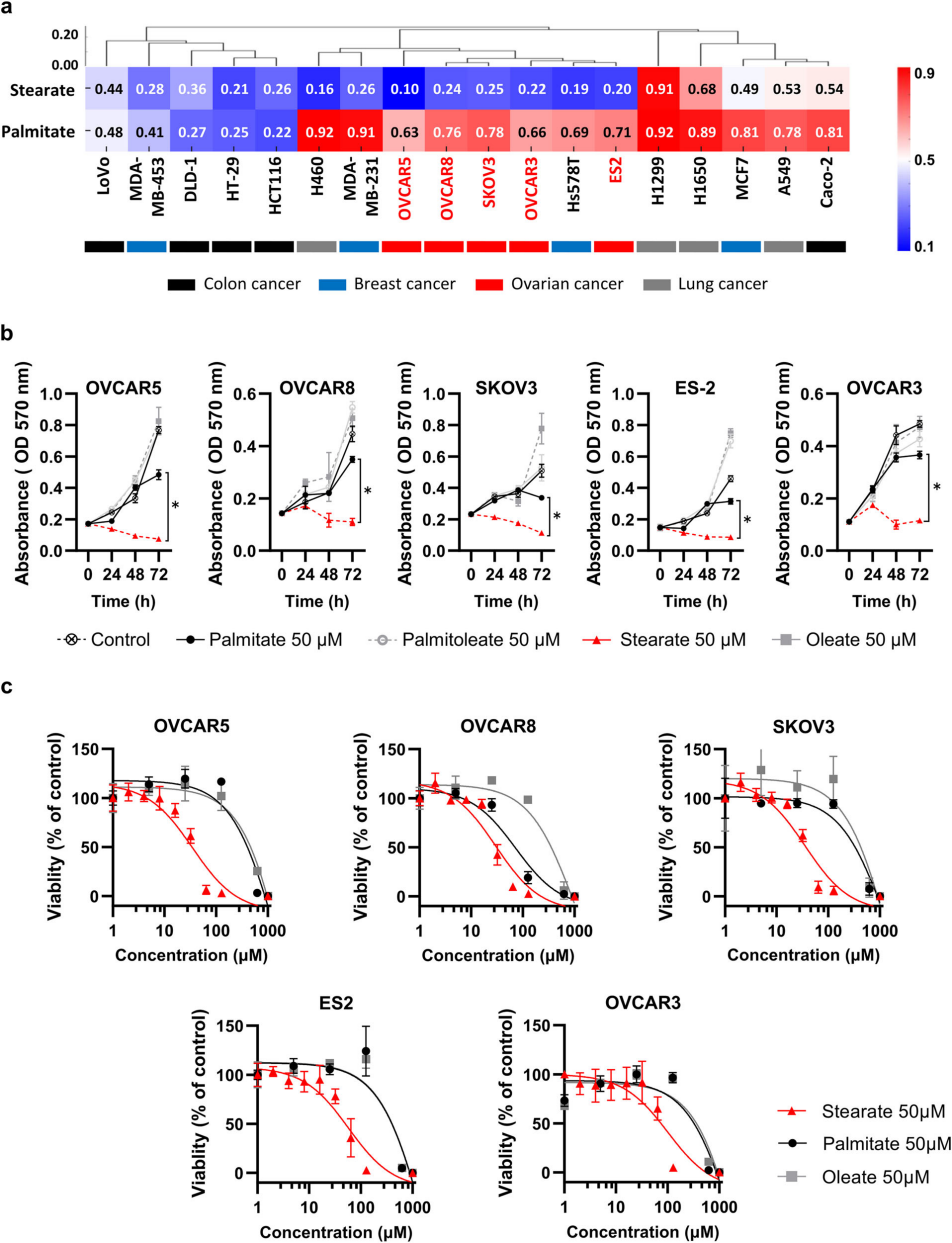
Different antiproliferative effects of stearate and palmitate on various cancer cell lines. **a** Clustering analysis of the results of the proliferation assay. Data from MTT proliferation assays of samples, including lung, breast, ovarian, and colon cancer cell lines, were subjected to k-means clustering analysis based on absorbance. The cells were cultured for 72 h and treated with 50 µM stearate or palmitate, followed by measurement using an MTT assay. The results were normalized to those of the fatty acid-free control. The ovarian cancer cell lines are highlighted in red (n = 6). **b** MTT assay of ovarian cancer cell lines. Ovarian cancer cell lines (OVCAR5, OVCAR8, SKOV3, ES-2, and OVCAR3) were treated with 50 µM free fatty acids (palmitate, palmitoleate, stearate, or oleate) for 72 h and then subjected to an MTT assay. Proliferation was assessed every 24 h. Data are presented as the means ± SEMs (n = 4; *p < 0.05, Mann–Whitney test). **c** Dose‒response curves for various fatty acids in different ovarian cancer cell lines. Representative dose‒response curves for stearate, palmitate, and oleate are shown based on quadruplicate data.

First, we evaluated the effects of palmitate and stearate on cellular function. Multiple human cancer cell lines (ovarian: OVCAR5, ES-2, SKOV3, OVCAR3, and OVCAR8; lung: H460, A549, H1650, and H1299; breast: Hs578T, MDA-MB0231, MDA-MB-453, and MCF7; and colorectal: LoVo, HCT116, HT290, DLD-1, and Caco-2) were cultured in palmitate- and stearate-supplemented media for 72 h, and the effects on cell proliferation was assessed. Overall, stearate inhibited cell growth to a greater extent than palmitate did across all cell lines (p = 0.000252, Wilcoxon matched-pairs test). Furthermore, k-means clustering analysis revealed that the cells could be segregated into three distinct groups based on their proliferative responses to the fatty acids: stearate ≈ palmitate, stearate > palmitate, and a group in which neither fatty acid influenced cell proliferation (Fig. 1a).

The ovarian cancer cell lines were exclusively categorized under the stearate > palmitate group, suggesting that a stronger inhibitory effect on ovarian cancer cell growth was observed with stearate than with palmitate. Additionally, similar experiments were conducted using the human mammary epithelial cell line MCF10A and the human ovarian surface epithelial cell line HOSE^20^. Despite both being immortalized normal cell lines, the sensitivity to stearate varied significantly between these two cell lines. The addition of stearate had a more pronounced effect on proliferation in HOSE cells than in MCF10A cells (p = 0.002165). Subsequent experiments focused primarily on ovarian cancer cell lines classified into the stearate > palmitate group to further investigate the potential anticancer effects of stearate.

Conversely, in culture media supplemented with monounsaturated fatty acids (MUFAs), palmitoleate and oleate did not markedly inhibit ovarian cancer cell growth. In fact, in certain cases, cell growth was enhanced, suggesting differential effects of SFAs and MUFAs on cancer cells (Supplementary Fig. 1a).

We conducted experiments using long-chain fatty acids that are frequently encountered in dietary and cellular contexts to identify which fatty acids significantly affect cell proliferation^7,21^. Ovarian cancer cell lines, namely, OVCAR5, OVCAR8, SKOV3, ES-2, and OVCAR3 cells, were treated with 50 µM palmitate, stearate, or oleate, and cell viability was measured after 24, 48, and 72 h. Stearate markedly impeded the growth of all cell lines beginning at 24 h (Fig. 1b). Dose–response curves measured after 72 h of exposure to each long-chain fatty acid revealed substantially lower IC_50_ values for stearate (36.96 ± 3.22 µM for OVCAR5 cells and 31.04 ± 1.97 µM for OVCAR8 cells) than for palmitate (1469.75 ± 74.61 µM for OVCAR5 cells and 74.97 ± 2.7 µM for OVCAR8 cells) (Fig. 1c, Supplementary Fig. 1b).

### Stearate induces apoptosis and DNA damage in ovarian cancer cells in vitro and in vivo

**Fig. 2:**
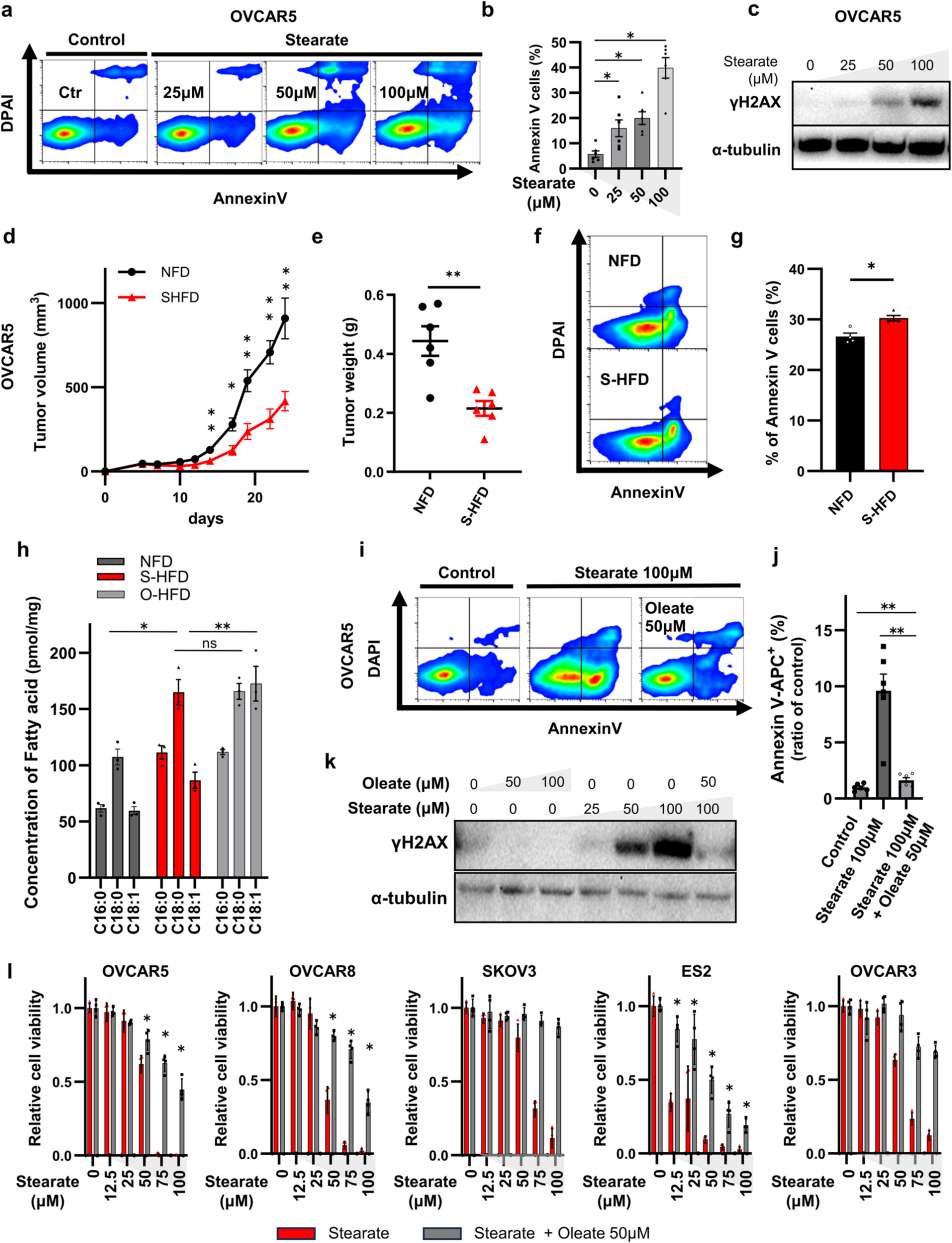
Stearate induces apoptosis and DNA damage in ovarian cancer cells in vitro and in vivo, whereas oleate mitigates stearate-induced cytotoxicity. **a**, **b** Flow cytometry image of stearate-induced apoptosis with Annexin V/PI staining and bar graphs. The apoptosis of OVCAR5 ovarian cancer cells treated with various stearate concentrations was analyzed using Annexin V/PI staining. Bar graphs (**b**) depict the percentage of Annexin V-positive cells compared with that in untreated controls, as determined using flow cytometry (**a**). The data are presented as the means ± SEMs (n = 4; *p < 0.05, Wilcoxon test). **c** Analysis of γH2AX expression using western blotting. The OVCAR5 cell line was treated with the indicated stearate concentrations for 24 h, and γH2AX expression was determined. α-Tubulin served as the loading control. **d–g** Xenograft mouse models harboring OVCAR5. Tumor growth curves (**d**) and tumor weights at collection (**e**). Data are presented as the means ± SEMs (n = 6; *p < 0.05, **p < 0.01, Mann–Whitney test). Apoptosis assay of tumor tissue using flow cytometry (**f**) and histograms (**g**) presenting the percentages of apoptotic cells (n = 4; *p < 0.05 and **p < 0.01, Mann–Whitney test). **h** Fatty acid levels in OVCAR5 xenografts assessed via LC‒MS. Fatty acid concentrations were quantified in tumors from mice fed the NFD, S-HFD, or O-HFD (identical to those in Fig. S9a, d–f). **i**, **j** Flow cytometry analysis of stearate-induced apoptosis with Annexin V/PI staining (**i**). The bar graph (**j**) shows the ratio of Annexin V-positive cells to control cells. Means ± SEMs (n = 6, **p < 0.01, ns; not significant, Wilcoxon matched-pairs test). **k** Analysis of γH2AX expression using western blotting. OVCAR5 cells were cultured for 24 h with the indicated concentrations of oleate and stearate, and γH2AX expression was analyzed. α-Tubulin served as a loading control. **l** Viability of various cell lines exposed to varying concentrations of stearate and oleate. The cells were treated with the indicated concentrations of stearate and coincubated with 50 µM oleate for 72 h. Cell viability, relative to that of the control cells, was assessed using an MTT assay (n = 4; *p < 0.05, Mann–Whitney test).

Next, we investigated whether stearate induced apoptosis similar to palmitate, as reported previously^12,14,16^. The flow cytometry analysis of Annexin V-positive cells revealed that stearate increased the apoptosis of OVCAR5 cells in a dose-dependent manner (Fig. 2a, b). As palmitate-induced apoptosis is related to DNA damage^22,23^, we examined whether stearate similarly affects OVCAR5 cells. A dose-dependent increase in γH2AX expression was observed following 24 h of stearate treatment (Fig. 2c). The findings were corroborated through comparative experiments utilizing OVCAR8 cells; flow cytometry and western blot assays were conducted to elucidate the effects of stearate on apoptosis and DNA damage, respectively (Supplementary Fig. 1c–e).

We conducted an in vivo study to obtain additional insights into our findings. We first used murine models to determine the impact of a HFD rich in oleate (O-HFD) on the growth of tumors derived from subcutaneously inoculated cancer cell lines (Supplementary Fig. 2a). Consistent with previous studies, tumor proliferation increased in mice harboring tumors derived from OVCAR5 cells that were fed an O-HFD (Supplementary Fig. 2b–e)^2,4,24^. Given the absence of notable disparities in body weight or blood insulin levels^25^ resulting from dietary variations, these findings prompted the hypothesis that the influence on tumor proliferation stemmed from the fatty acids themselves rather than alterations in the physiological conditions of the mice. Initially, the fat in the O-HFD group was mainly composed of oleate (64.3%), with a low stearate content (7.5%; Supplementary Table 1).

Next, we investigated the effects of an S-HFD with a significantly greater per-calorie stearate content (33.35%; Supplementary Table 1). Importantly, the S-HFD group exhibited a significant reduction in tumor growth compared with the NFD group (Fig. 2d, e). Flow cytometry and IHC revealed a greater degree of tumor cell apoptosis in the S-HFD group than in the NFD group (Annexin V-positive rate: 26.6% vs. 30.27%, cleaved caspase-3-positive area: 0.56% vs. 1.0%; Fig. 2f, g; Supplementary Fig. 2f, g). IHC was performed for γH2AX to evaluate DNA damage, and the results revealed a significant increase in the proportion of γH2AX-positive cells in the S-HFD group compared with that in the NFD group (H score: 38.2 vs. 57.5; Supplementary Fig. 2f, h). These findings were validated using SKOV3 cells (Supplementary Fig. 2i–k). No significant differences in body weight or vital organs, including the liver, kidneys, and colorectal epithelial cells, were observed between the S-HFD and NFD groups (Supplementary Fig. 3a–c). These data collectively demonstrate that stearate induces cytotoxicity, DNA damage, and apoptosis in ovarian cancer cells.

### Oleate mitigates stearate-induced cytotoxicity

Long-chain fatty acids can be converted to other long-chain fatty acids in biological systems^26^. Therefore, we altered the dietary long-chain fatty acid composition to generate an S-HFD and evaluated whether these changes were reflected in the tumor tissue. The S-HFD group had a significantly higher tumor stearate content than the NFD group (107.3 vs. 164.9 pmol/mg, p = 0.012094). Notably, stearate levels were elevated even in the O-HFD group, almost matching those in the S-HFD group (O-HFD vs. S-HFD: 164.9 vs. 165.7 pmol/mg, p = 0.957080). However, oleate levels were higher in the O-HFD group than in the S-HFD group (O-HFD vs. S-HFD: 172.5 vs. 86.62 pmol/mg, p = 0.007269; Fig. 2h). These findings led us to propose that oleate may mitigate the tumor-suppressive effects of stearate. Oleate ameliorates palmitate-induced endoplasmic reticulum (ER) stress and DNA damage^17,23,27–29^; therefore, we next examined whether similar phenomena occurred in our study.

We first investigated the effect of oleate on tumor apoptosis. Stearate-induced apoptosis was significantly reduced following the addition of 50 µM oleate to OVCAR5 and OVCAR8 cell (Fig. 2i, j; Supplementary Fig. 4a, b). Moreover, the addition of 50 µM oleate almost completely abrogated the stearate-induced increase in γH2AX expression in OVCAR5 and OVCAR8 cells (Fig. 2k, Supplementary Fig. 4c), resulting in reduced cytotoxicity. We treated OVCAR5, OVCAR8, SKOV3, ES-2, and OVCAR3 cells with varying concentrations of stearate in the presence of 50 µM oleate (Fig. 2l). The addition of 50 µM oleate significantly ameliorated the stearate-induced decrease in cell viability. We next explored the effects of oleate on stearate-induced cell death. Oleate rescued the cells incubated with 100 µM stearate from death in a concentration-dependent manner (Supplementary Fig. 4d). In OVCAR5 cells, the addition of 25 µM oleate rescued cell proliferation to levels almost comparable to those achieved with the addition of 100 µM oleate. Overall, oleate attenuates stearate-induced cytotoxic effects, including DNA damage and apoptosis, on ovarian cancer cells.

### The inhibition of unsaturation increases stearate toxicity

**Fig. 3:**
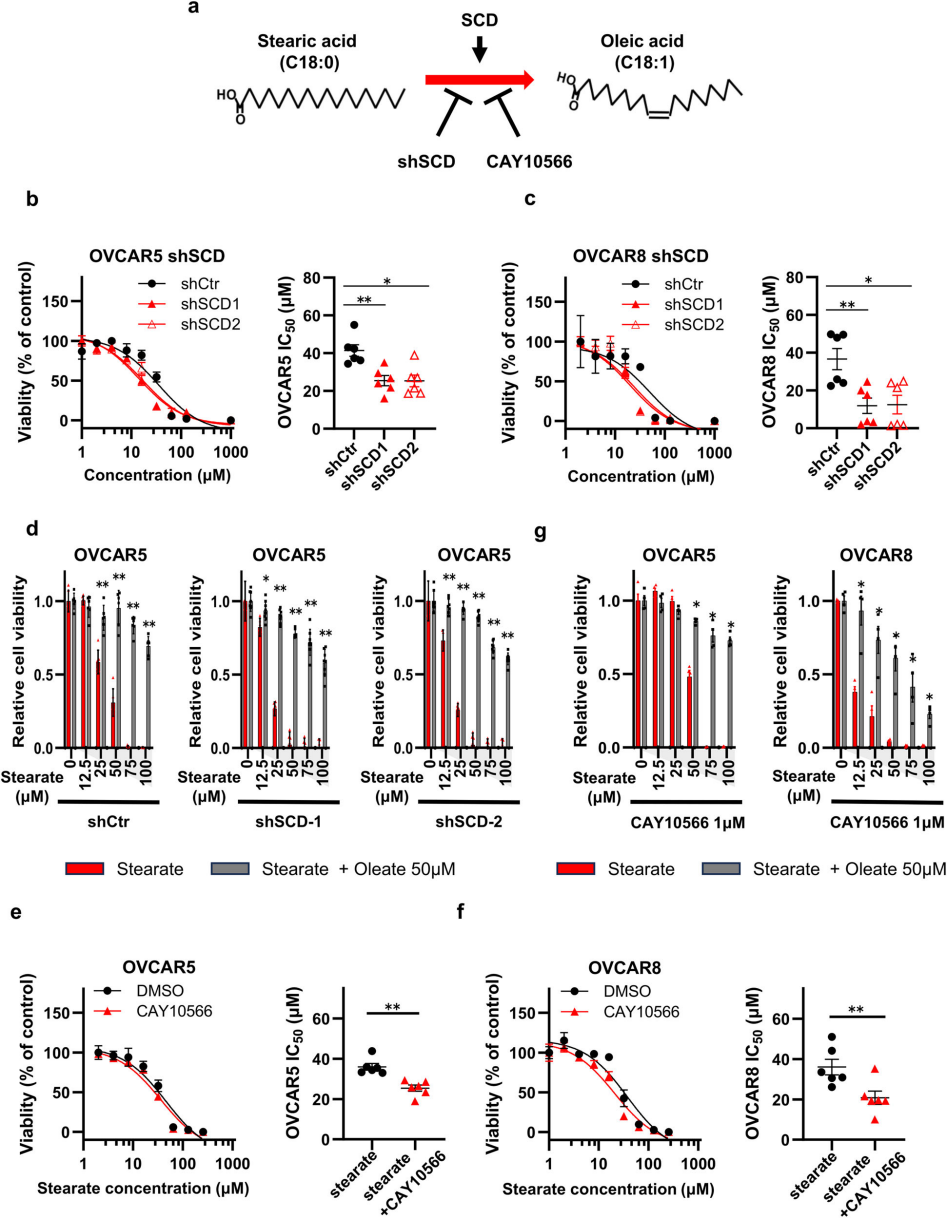
The inhibition of unsaturation increases stearate toxicity, and exogenous oleate mitigates it. **a** Proposed functional mechanism of SCD in stearate metabolism. shRNA-mediated knockdown and CAY10566 were used to inhibit SCD, the enzyme that converts stearate to oleate. **b** IC_50_ values of stearate in shSCD-treated OVCAR5 cells. SCD knockdown lowered the IC_50_ (n = 6; *p < 0.05 and **p < 0.01, Mann–Whitney test). **c** Analogous IC_50_ findings in shSCD-treated OVCAR8 cells (n = 6; *p < 0.05 and **p < 0.01, Mann–Whitney test). **d** Effects of oleate on the viability of stearate-treated shSCD-transfected OVCAR5 cells. The cells were treated with the indicated concentrations of stearate and coincubated with 50 µM oleate for 72 h; viability was measured via an MTT assay (n = 6; *p < 0.05 and **p < 0.01, Mann–Whitney test). IC_50_ values of stearate in OVCAR5 (**e**) and OVCAR8 (**f**) cells treated with 1 μM CAY10566 or the DMSO control (n = 6; **p < 0.01, Mann–Whitney test). **g** Cell viability in response to treatment with stearate and oleate in the presence of 1 μM CAY10566. The cells were treated with the indicated concentrations of stearate and coincubated with 50 µM oleate for 72 h; viability was assessed after 72 h via an MTT assay (n = 6; *p < 0.05, Mann–Whitney test).

In biological systems, stearate is converted to oleate by stearoyl-CoA desaturase 1^30^ (SCD1; Fig. 3a). SCD1 overexpression has been documented in various cancers, including ovarian cancer^31–33^. Li et al. ^8^ argued that the malignancy of high-grade serous ovarian cancer (HGSC) is significantly influenced by the endogenous oleate produced by SCD1. Therefore, we explored the involvement of endogenous and exogenous oleate in cancer pathogenesis. We transduced shRNA sequences targeting SCD1 (shSCD-1 and shSCD-2) to inhibit endogenous oleate synthesis or a control shRNA (shCtr) into OVCAR-5 and OVCAR-8 cells (Supplementary Fig. 5a–d).

The inhibition of SCD1 led to a marked increase in cellular sensitivity to stearate (Fig. 3b, c). Conversely, the addition of oleate substantially ameliorated stearate-induced cytotoxicity (Fig. 3d, Supplementary Fig. 5e, f). We then overexpressed SCD1 by introducing an open reading frame (Supplementary Fig. 5g) and examined whether the toxicity of stearate was altered. The results indicated that the toxicity of stearate was significantly attenuated (Supplementary Fig. 5h). Furthermore, we conducted additional experiments using an SCD1 inhibitor (CAY10566). The incubation with 1 µM CAY10566^34,35^ did not inhibit cell proliferation; however, the concentrations of stearate and oleate in the OVCAR5 cells were significantly altered (Supplementary Fig. 6a–c). Similar to SCD knockdown (SCD-KD), the addition of 1 µM CAY10566 increased the cellular sensitivity to stearate (Fig. 3e, f), whereas the growth-inhibitory effect was significantly mitigated by the addition of oleate (Fig. 3g). These trends were consistent across other cell lines, including SKOV3, ES2, and OVCAR3 cells (Supplementary Fig. 6d–f).

These results indicate that the inhibition of SCD1 enzymatic activity increases sensitivity to stearate due to the lack of endogenous oleate production. Moreover, we demonstrate that sufficient exogenous oleate supplementation significantly mitigates the toxicity of stearate, even under such conditions.

### Stearate induces cytotoxicity via ER stress and CHOP activation

**Fig. 4:**
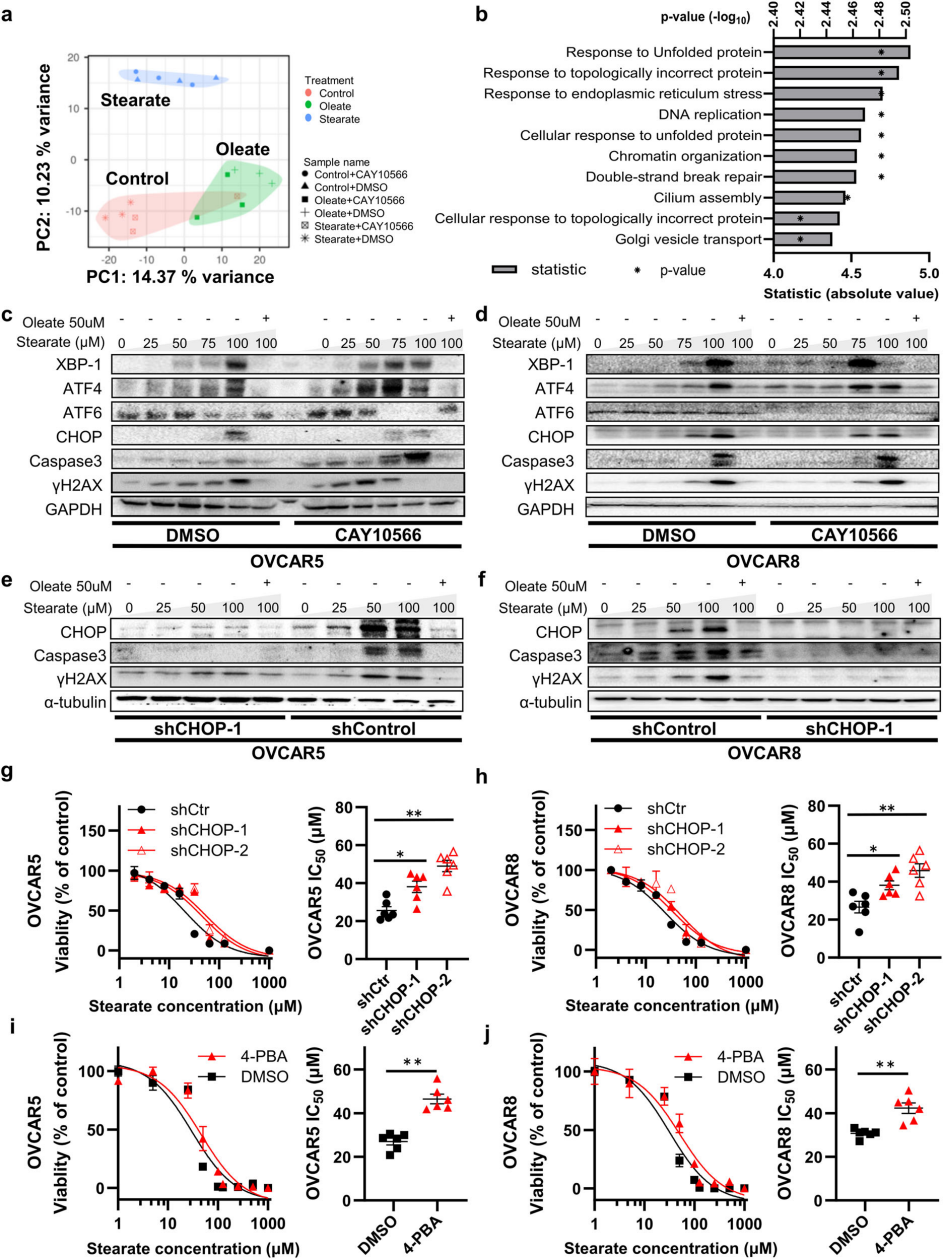
Stearate induces cytotoxicity via ER stress and CHOP activation. **a** RNA-Seq of OVCAR5 cells subjected to various treatments. The cells were treated and cultured for 24 h before RNA-seq. Principal component analysis revealed distinct gene expression profiles without treatment-based separation. **b** Top 10 functionally enriched terms. The biological processes induced by stearate compared with those induced by DMSO are shown. Representative western blot analysis of proteins involved in the unfolded protein response (UPR), apoptosis, and DNA damage in OVCAR5 (**c**) and OVCAR8 (**d**) cells treated with the indicated concentrations of stearate and oleate. GAPDH was used as an internal control. Representative western blot analysis of protein expression following the knockdown of CHOP (shCHOP) in OVCAR5 (**e**) and OVCAR8 (**f**) cells. α-Tubulin was used as an internal control. IC_50_ values of stearate in shCHOP-transfected OVCAR5 (**g**) and OVCAR8 (**h**) cells (n = 6; *p < 0.05 and **p < 0.01, Mann–Whitney test). IC50 values of stearate in OVCAR5 (**i**) and OVCAR8 (**j**) cells treated with 5 μM 4-PBA or the DMSO control (n = 6; **p < 0.01, Mann–Whitney test).

Next, we sought to elucidate the mechanisms underlying stearate-mediated cytotoxicity. We treated OVCAR5 cells with (i) DMSO, (ii) 1 µM CAY10566, (iii) 50 µM stearate+DMSO, (iv) 50 µM stearate+1 µM CAY10566, (v) 50 µM oleate+DMSO, or (vi) 50 µM oleate+1 µM CAY10566 and performed an RNA sequencing analysis. Principal component analysis (PCA) revealed that the presence or absence of stearate strongly contributed to PC1, whereas the presence or absence of oleate influenced PC2. However, 1 µM CAY10566 had limited effects (Fig. 4a).

Gene Ontology analysis revealed 643 differentially expressed genes (DEGs), of which 401 were upregulated and 242 were downregulated between 50 µM stearate-treated and control OVCAR5 cells (false discovery rate [FDR] < 0.05, minimum fold change>1.25; Supplementary Fig. 7a, b). The top 10 significantly upregulated DEGs were enriched in Gene Ontology terms associated with the unfolded protein response (UPR) and ER stress in 50 µM stearate-treated OVCAR5 cells compared with control cells (Fig. 4b; FDR < 0.05).

The UPR involves the ATF6, IRE1α, and PERK pathways^36^. Our western blot analysis confirmed that stearate induced the concentration-dependent activation of UPR-related proteins, including ATF6 and XBP-1, which are downstream transcription factors of IRE1α, and ATF4, which is a downstream transcription factor of PERK. Moreover, the levels of the proapoptotic transcription factor CHOP^37^ and the apoptotic markers cleaved caspase-3 and γH2AX were upregulated (Fig. 4c, d).

We further examined whether the addition of oleate mitigated the activation of ER stress response pathways. The activation of ER stress response pathways was negated by the addition of 100 µM oleate to OVCAR5 and OVCAR8 cells (Fig. 4c, d). Furthermore, the addition of 1 µM CAY10566 enhanced the stearate-dependent activation of the UPR-related proteins CHOP, cleaved caspase-3, and γH2AX; however, this activation of the UPR pathway was almost completely abrogated by exogenous oleate (Fig. 4c, d).

Long-term exposure to mild ER stress or short-term exposure to severe ER stress induces CHOP-mediated apoptosis^13,38^. We generated CHOP-knockdown OVCAR5 and OVCAR8 cell lines via lentiviral infection of the CHOP shRNA to explore whether stearate induced apoptosis via CHOP (Supplementary Fig. 7c, d).

Following the inhibition of CHOP expression, the levels of cleaved caspase-3 and γH2AX, which were increased in a concentration-dependent manner by stearate treatment, were significantly reduced (Fig. 4e, f; Supplementary Fig. 7e, f). Moreover, CHOP knockdown significantly enhanced resistance to stearate-induced cytotoxicity (Fig. 4g, h). Furthermore, we investigated the effect of adding 4-PBA, a UPR inhibitor. In both the OVCAR5 and OVCAR8 cell lines, the addition of 4-PBA suppressed the expression of CHOP, even under stearate treatment (Supplementary Fig. 7g, h), and simultaneously, resistance to stearate-induced cytotoxicity was significantly increased (Fig. 4i, j). These results indicate that stearate-induced cytotoxicity is mediated by ER stress and CHOP activation. As shown above, we confirmed that stearate sensitivity varies among cell lines (Fig. 1a). We then investigated whether these differences were due to variations in the degree of ER stress response pathway activation. In MCF10A cells (stearate-nonresponsive cells), we observed minimal CHOP induction by stearate, which differed significantly from the findings for HOSE and OVCAR5 cells (stearate-responsive cells) (Supplementary Fig. 7i, j). In H1299 cells (stearate-nonresponsive cells), we detected constant CHOP expression regardless of the addition of stearate, which was not decreased by oleate. These findings also significantly differed from those in HOSE and OVCAR5 cells (Supplementary Fig. 7i, j).

Overall, in cell lines such as ovarian cancer cells, which exhibit high cytotoxicity to stearate, exogenous stearate activated ER stress response pathways, induced DNA damage, and inhibited the proliferation of cancer cells. Consistently, the addition of exogenous oleate attenuated the ER stress response pathway activated by stearate, reducing stearate toxicity in ovarian cancer.

### Differential cellular responses to palmitate and stearate

In our previous findings, stearate displayed more pronounced cytotoxicity in a variety of cell lines than palmitate did. We conducted a comprehensive analysis to further explore the differential effects of these two fatty acids on cellular processes.

**Fig. 5:**
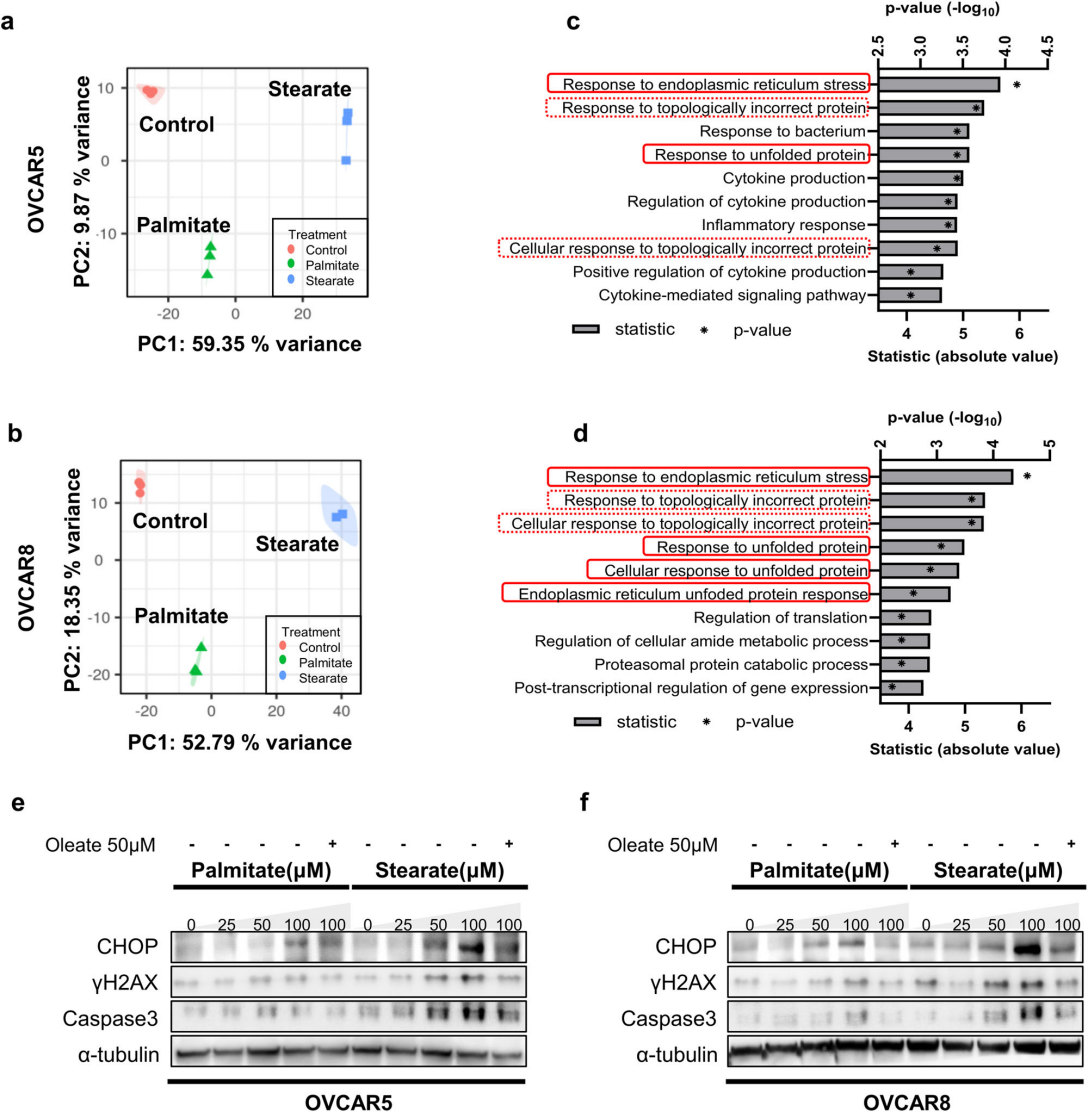
Differential cellular response to palmitate and stearate. RNA-seq followed by principal component analysis of OVCAR5 cells (**a**) and OVCAR8 cells (**b**). The cells were cultured for 24 h under different conditions (control, 50 µM palmitate or 50 µM stearate) before RNA extraction. **c**, **d** Analysis of GO terms. The top 10 functionally enriched pathways in the OVCAR5 cells (**c**) and OVCAR8 cells (**d**) are shown. Pathways related to the UPR are circled in red, and pathways associated with incorrectly folded proteins are circled in red dotted lines. Representative images of western blot analyses of OVCAR5 cells (**e**) and OVCAR8 cells (**f**). The cells were cultured for 24 h with various concentrations of palmitate or stearate in the presence or absence of oleate, as shown. The levels of CHOP, γH2AX and cleaved caspase3 were analyzed. α-Tubulin was used as a loading control.

Three groups were established for each of the OVCAR5, OVCAR8, and SKOV3 cell lines: a 50 μM stearate treatment group, a 50 μM palmitate treatment group, and a control group. RNA sequencing followed by principal component analysis (PCA) were subsequently performed to examine the differences in gene expression among the three groups (Fig. 5a, b; Supplementary Fig. 8a). For all three cell lines, the greatest difference was observed between the stearate treatment group and the combined palmitate and control groups. Gene Ontology (GO) analysis of the DEGs identified between the stearate and palmitate groups revealed that, across all three cell lines examined, the top 10 significantly upregulated GO terms were enriched in pathways associated with the UPR, ER stress, and responses to topologically incorrect proteins (Fig. 5c, d; Supplementary Fig. 8b).

These in silico analyses suggest that palmitate and stearate exert significantly different effects on cells, with particularly notable differences observed in the ER pathway. We then investigated the responses of OVCAR5 and OVCAR8 cells to stearate and palmitate. Notably, palmitate induced CHOP expression in these cells but to a lesser extent than stearate did. Additionally, the activation of cleaved caspase-3 by palmitate was less pronounced than that induced by stearate (Fig. 5e, f). Furthermore, we examined various cancer cell lines derived from different tissues (Supplementary Fig. 8c). In MDA-MB-231, Hs578T, and H460 cells, in which stearate cytotoxicity was greater than that of palmitate, stearate induced CHOP expression more strongly than palmitate did, and this induction was attenuated by oleic acid. We also examined LoVo and DLD-1 cells, in which stearate and palmitate cytotoxicity was similar. In LoVo cells, CHOP was strongly expressed even under control conditions, making it difficult to evaluate the effects of palmitate or stearate. However, in DLD-1 cells, stearate induced CHOP expression more strongly than palmitate did. We further examined A549, H1299, and Caco2 cells, which exhibited low cytotoxicity of both palmitate and stearate. While neither stearate nor palmitate induced CHOP expression in Caco2 and H1299 cells, CHOP expression was induced in A549 cells, with stearate inducing a stronger response than palmitate. Moreover, the induction of CHOP expression by stearate was attenuated by the addition of oleic acid to A549 cells.

These results suggest that stearate and palmitate exert distinct effects on a wide range of cancer cell lines, with significant differences observed in the induction of the ER stress response.

### The inhibition of unsaturation along with dietary supplementation with stearate hinders tumor growth, which is reversed by oleate supplementation

**Fig. 6:**
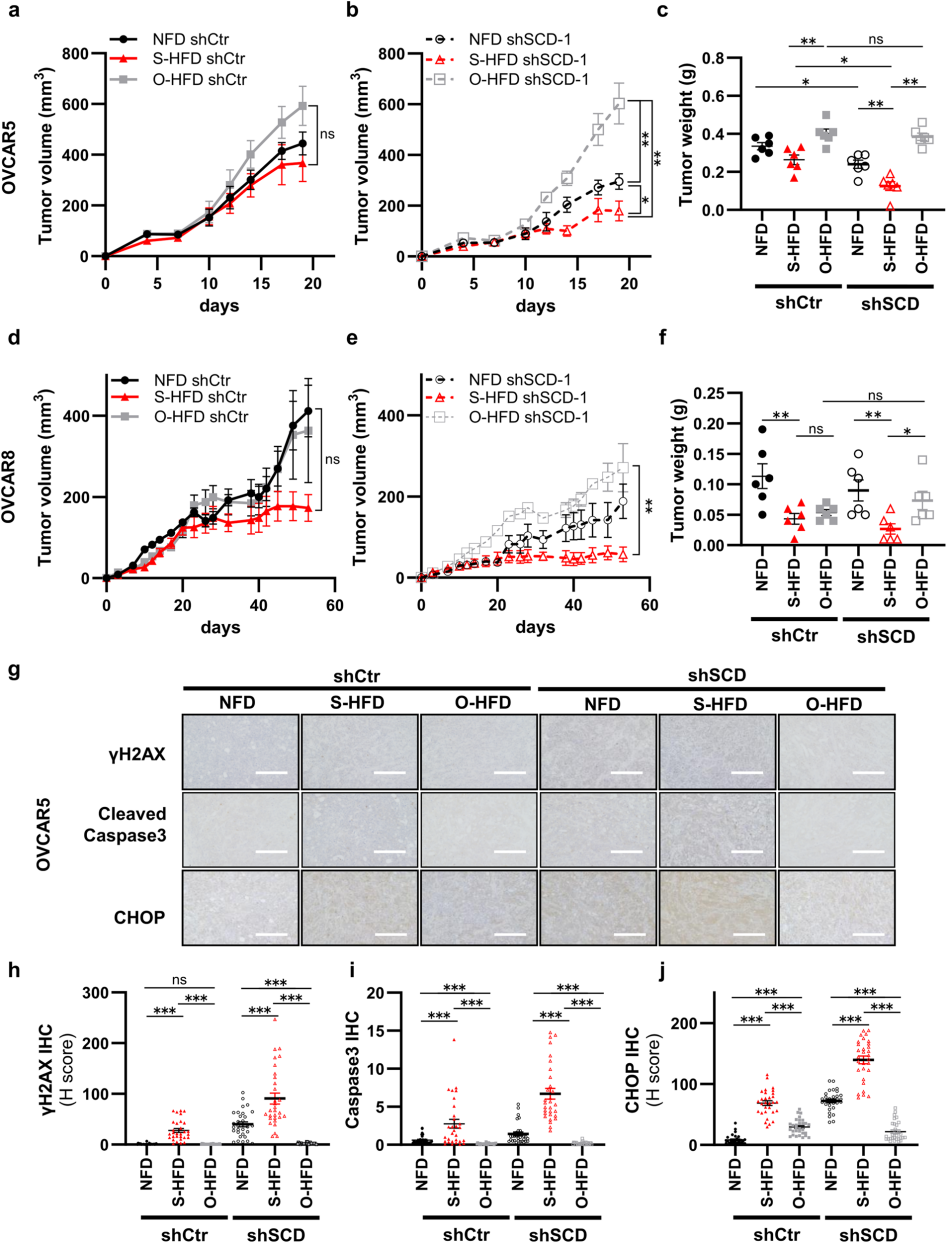
The inhibition of unsaturation along with dietary supplementation with stearate hinders tumor growth, which is reversed by the addition of oleate. Tumor growth in mice following subcutaneous injection of control (shCtr) (**a**) or SCD-knockdown (shSCD) (**b**) OVCAR5 cells. The mice were divided into three dietary groups, namely, the normal-fat diet (NFD), O-HFD, and S-HFD groups, and received the intervention from 3 days before injection until the end of the study (n = 6). The data are presented as the means ± SEMs. *p < 0.05, **p < 0.01, and ns stands for not significant; the Mann–Whitney test was applied between shCtr samples and the Wilcoxon matched-pairs signed rank test was applied between shCtr and shSCD-1 pairs. **c** Weights of the tumors at the end of the experimental period. **d–f** Validation of the results using OVCAR8 cells (n = 6). The data are presented as the means ± SEMs. *p < 0.05, **p < 0.01, and ns stands for not significant; the Mann–Whitney test was applied between shCtr samples and the Wilcoxon matched-pairs signed rank test was applied between shCtr and shSCD-1 pairs. **g** Representative images of immunohistochemical staining of tumor tissues derived from shCtr-OVCAR5 cells or shSCD-1-OVCAR5 cells depicting the levels of cleaved caspase-3, γH2AX, and CHOP. **h–j** Quantitative analysis of cleaved caspase-3, γH2AX, and CHOP levels in tissue. (n = 30; ***p < 0.001, ns: not significant, Mann–Whitney test).

We fed mice an S-HFD, O-HFD, or NFD to validate our results in vivo (Supplementary Fig. 9a–c). In the S-HFD group, which was subcutaneously injected with SCD1-knockdown (SCD1-KD) OVCAR5 cells, tumor growth was significantly inhibited compared with that in the NFD group (SCD1-KD and S-HFD vs. SCD1-KD and NFD; 0.125 g vs. 0.240 g, p = 0.006494; Fig. 6b, c). Conversely, the O-HFD group displayed significantly greater tumor growth than the S-HFD and NFD groups (Fig. 6c). In experiments using sh-control cell lines, the S-HFD group exhibited stronger growth suppression than the NFD and O-HFD groups, although this trend was less pronounced than that observed in experiments using SCD1-KD cells (sh-control and S-HFD vs. sh-control and O-HFD; 0.2633 g vs. 0.4017 g, p = 0.006494; Fig. 6a, c). Furthermore, no significant differences in tumor growth were observed between sh-control and SCD1-KD cells in the O-HFD group (Fig. 6c).

In the S-HFD group subcutaneously injected with SCD1-KD OVCAR8 cells, the greatest tumor growth suppression was noted, with a significant difference compared with that in the O-HFD group (SCD1-KD and S-HFD vs. SCD1-KD and O-HFD; 0.02667 g vs. 0.0733 g, p = 0.019481; Fig. 6d–f). The same trend was observed when the animals were injected with the sh-control cell line; however, no significant differences were observed between the S-HFD and O-HFD groups (sh-control and S-HFD vs. sh-control and O-HFD: 0.0433 g vs. 0.0533 g, p = 0.4848). Additionally, no significant differences in tumor growth were detected between mice injected with sh-control or SCD1-KD cells and those fed the O-HFD, as observed with OVCAR5 cells (Fig. 6f).

Next, we examined whether the UPR pathway, DNA damage, and apoptosis were modulated in vivo. IHC of OVCAR5 cell-derived tumors revealed the marked upregulation of CHOP expression in the S-HFD group and the most significant upregulation in the SCD1-KD group (Fig. 6g–j). Conversely, CHOP expression was almost completely abrogated in the O-HFD group, regardless of whether sh-control or SCD1-KD cells were injected. We also assessed γH2AX and cleaved caspase-3 levels and observed trends consistent with those of CHOP expression. Similar results were obtained using OVCAR8 cells (Supplementary Fig. 9d–g).

We conducted additional experiments using CAY10566 (Supplementary Fig. 10a–c). In mice injected with OVCAR5 and OVCAR8 cells, the CAY10566-treated groups exhibited more significant tumor growth suppression when fed the S-HFD than when fed the NFD or O-HFD (Supplementary Fig. 10d–f and 11a–c). In the vehicle-treated group, the mice fed the S-HFD exhibited the lowest tumor growth, but this trend was less pronounced than that in the CAY10566 group. Moreover, no significant differences were observed between the vehicle and CAY10566 groups when they were fed the O-HFD. The levels of γH2AX and cleaved caspase-3 were most significantly increased in the CAY10566 + S-HFD group, whereas almost no expression was observed in the O-HFD groups, irrespective of whether mice were in the vehicle or CAY10566 group (Supplementary Fig. 10g–j and 11d–g). Assessments of stearate and oleate concentrations within tumor tissues revealed an increase in the stearate concentration of 1.5- to 2-fold in the S-HFD-, O-HFD-, or CAY10566-administered group compared with that in the NFD+vehicle group. Despite the administration of CAY10566, the O-HFD increased oleate levels by approximately 1.5-fold, which correlated with increased proliferation in the tumors. Conversely, S-HFD in combination with CAY10566 administration resulted in a significant increase in stearate levels to 185 pmol/mg while maintaining oleate levels at 50 pmol/mg, which was lower than that in the NFD-vehicle group, thus exerting a pronounced inhibitory effect on tumor growth (Supplementary Fig. 11h).

Overall, robust tumor-suppressive effects were achieved in vivo by increasing tumor stearate levels via S-HFD feeding coupled with oleate inhibition mediated by SCD inhibition. Additionally, the excessive intake of oleate through the O-HFD significantly diminished this effect.

### Supplementation with stearate, along with the inhibition of unsaturation, have significant antiproliferative effects on ovarian cancer patient-derived xenograft (PDX) models

**Fig. 7:**
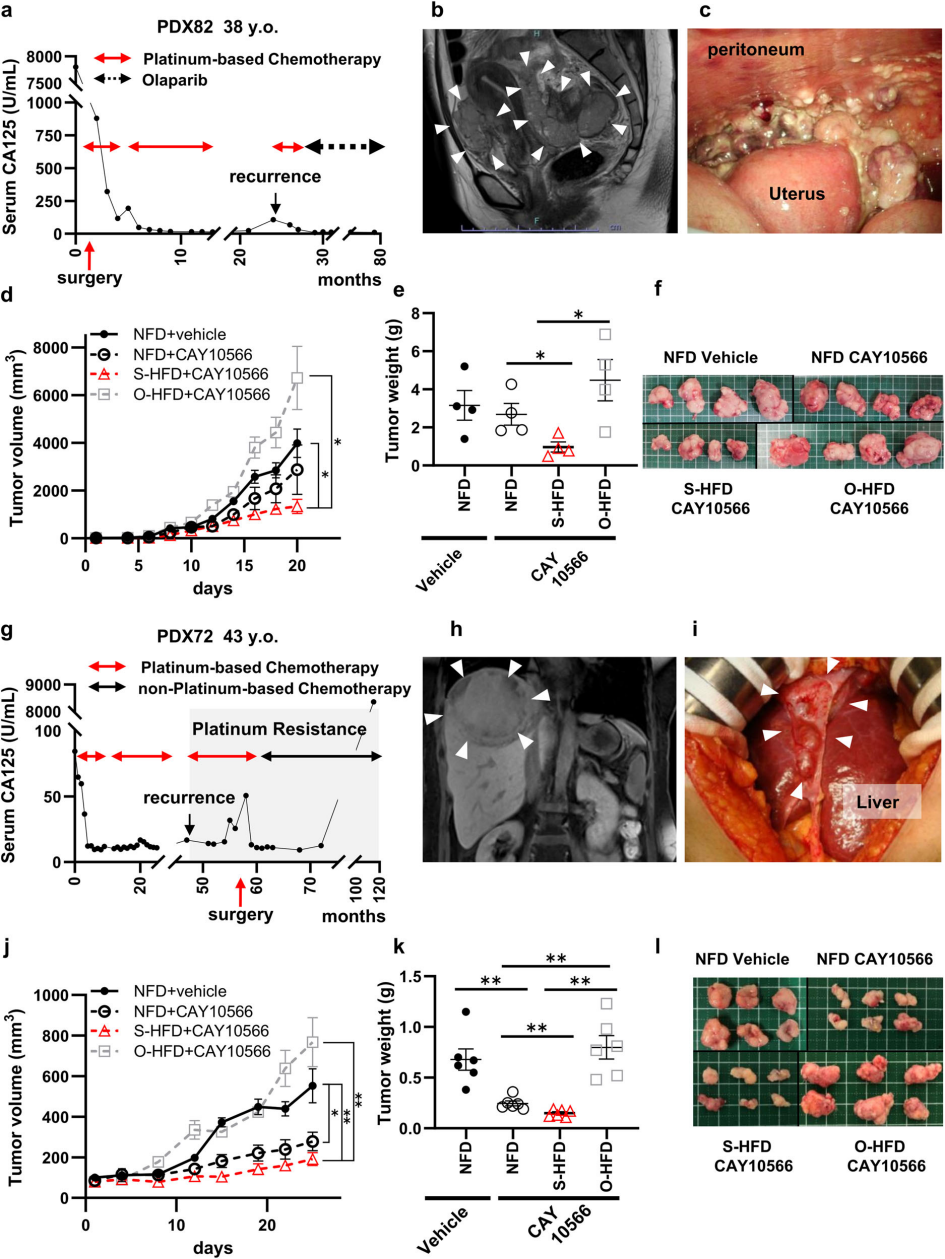
Supplementation with stearate, along with the inhibition of unsaturation, have significant antiproliferative effects on ovarian cancer patient-derived xenograft (PDX) models. **a** Longitudinal assessment of serum CA-125 levels during therapeutic intervention in a patient with high-grade serous ovarian carcinoma (source of PDX82). **b** Magnetic resonance imaging (MRI) of a 38-year-old woman (source of PDX82). The sagittal T2-weighted MR image highlights the tumor mass; the white arrow indicates the tumor. **c** Diagnostic laparoscopy reveals the frozen pelvis phenomenon due to significant tumor occupation in the pelvic cavity. **d** PDX82 proliferation in a mouse model under various nutritional and CAY10566 treatment conditions (n = 4; *p < 0.05, Mann–Whitney test). **e** End-point tumor mass in mice harboring PDX82 (n = 4; *p < 0.05, **p < 0.01, Mann–Whitney test). **f** PDX82 tumor specimens. Representative images from each condition are shown. **g** Longitudinal assessment of serum CA-125 levels during therapeutic intervention in a patient with high-grade serous ovarian carcinoma (source of PDX72). **h** MRI of a platinum-resistant tumor in a 43-year-old woman (source of PDX72); the coronal T2-weighted MR image highlights the tumor mass, and the white arrow indicates the tumor. **i** Intraoperative abdominal image captured during hepatic metastatic tumor resection. **j** PDX72 tumor proliferation in a mouse model under various nutritional and CAY10566 treatment conditions (n = 6; *p < 0.05 and **p < 0.01, Mann–Whitney test). **k** End-point tumor mass in the PDX72 model (n = 6; **p < 0.01, Mann–Whitney test). **l** PDX72 tumor specimens; images from each experimental group are shown.

We next conducted experiments using patient-derived xenograft (PDX) models to evaluate the applicability of our findings in a clinical setting. Conducting large-scale interventions to assess the effects of dietary changes is challenging; however, drug responses in PDXs have been suggested to correlate with patients’ clinical outcomes^39^. Therefore, we utilized two PDXs from distinct clinical backgrounds (PDX72 and PDX82; Supplementary Texts) that were established from patients treated at our institution. PDX82 was sourced from a 38-year-old female patient with stage IIIC HGSC harboring a *BRCA2* mutation. This patient was sensitive to platinum-based chemotherapy and maintained no long-term evidence of disease under poly(ADP‒ribose) polymerase inhibitor (PARPi)^40,41^ treatment (Fig. 7a–c, Supplementary Fig. 12a–c). In PDX82 experiments, while treatment with CAY10566 alone had limited effectiveness, tumor growth was significantly inhibited when these mice were fed an S-HFD (NFD-CAY10566: 2685 mg vs. S-HFD-CAY10566: 970 mg, p = 0.0285; Fig. 7d–f). However, the O-HFD led to significantly larger tumors than did the S-HFD, even with CAY10566 administration (S-HFD-CAY10566: 970 mg vs. O-HFD-CAY10566: 970 mg, p = 0.0285).

Another PDX, PDX72, was derived from a 43-year-old woman who developed platinum-resistant recurrent HGSC. The tumors were collected during secondary debulking surgery. Despite surgery, the patient quickly experienced a relapse, and neither platinum-based chemotherapy nor anti-VEGF antibodies^42^ were effective, resulting in a poor prognosis (Fig. 7g–i, Supplementary Fig. 13a–c). Studies using PDX72 revealed that CAY10566 administration alone inhibited tumor growth, and this effect was further enhanced by feeding the S-HFD to mice (NFD-vehicle: 678.3 mg vs. NFD-CAY10566: 245.0 mg vs. S-HFD-CAY10566: 150 mg, p = 0.0021 and 0.0043, respectively; Fig. 7j–l). However, despite CAY10566 treatment, mice fed the O-HFD developed significantly larger tumors than those fed the S-HFD (S-HFD-CAY10566: 150 mg vs. O-HFD-CAY10566: 798.3 mg, p = 0.0021).

The results of the IHC analysis of these two PDX models in terms of the UPR, DNA damage, and apoptosis markers were consistent; the highest levels of CHOP, γH2AX, and cleaved caspase-3 were observed in the CAY10566 + S-HFD group, whereas the levels of these markers were significantly reduced in the O-HFD group (Supplementary Fig. 12d–g and 13d–g).

Overall, the combined administration of CAY10566 and S-HFD significantly suppressed tumor growth in two distinct PDX models with different clinical backgrounds and outcomes. Furthermore, even in cases sensitive to CAY10566 alone, tumor proliferation was enhanced when the O-HFD was consumed, suggesting that the antitumor effect of CAY10566 can be compromised by an O-HFD.

## Discussion

In this study, we extensively explored the various effects of long-chain fatty acids on cancer cell proliferation. Studies using multiple organ-derived cancer cells have revealed that SFAs, known for their lipotoxicity in normal cells^43^—specifically palmitate and stearate—exert inhibitory effects on the growth of cancer cell lines. Notably, stearate exhibited an antiproliferative effect on a broader range of cancer cells than palmitate did. Detailed investigations revealed significant differences between stearate and palmitate, particularly in the induction of the UPR pathway. These findings suggest that the greater cancer cell growth-inhibitory effect of stearate than that of palmitate is attributable to this differential activation of the UPR. Specifically, palmitate showed limited efficacy in several cell lines, whereas stearate was more potent, with all six ovarian cancer cell lines included in this study falling into this category. Furthermore, the normal human ovarian surface epithelial cell line (HOSE) was strongly affected by stearate, a result that differed significantly from that of the normal human mammary epithelial cell line (MCF10A). Given the variable effects of long-chain fatty acids across different tissue types^5^, this finding suggests that ovarian tissues might possess heightened susceptibility to the cytotoxic effects of stearate, and this sensitivity could be extended to ovarian cancers, although the detailed mechanisms remain unclear.

Our findings demonstrated that stearate induced DNA damage and apoptosis through the dose-dependent activation of the UPR pathway. This phenomenon was directly mitigated by oleate, and impeding the conversion of stearate to oleate amplified the cytotoxic effects of stearate. Wieder et al. ^5^ segregated long-chain fatty acid-elicited cellular damage into two major pathways, the UPR and ROS generation, and revealed that the detrimental effects associated with the UPR could be reversed by oleate treatment. Our results corroborate these findings. Moreover, our findings establish that the S-HFD, along with an SCD inhibitor, exerted the most potent antiproliferative effects by accumulating stearate and limiting oleate in mice harboring xenografts derived from various cancer cell lines. These findings provide evidence that dietary modifications can induce the accumulation of excess stearate and limit the oleate content in tumors, thus inhibiting tumor growth. To our knowledge, this study is the first to clarify the strong therapeutic effect of excess dietary intake of stearate and limited intake of oleate on cancers. Detailed analyses of dietary lipid modifications, such as palmitate supplementation, in previous studies have also shown that stearate levels increase significantly^17,44^. Combined with our results, a substantial increase in the level of stearate, but not palmitate, is essential for eliciting antitumor effects.

HGSC is the predominant histological subtype of ovarian cancer^45^ and is often diagnosed at advanced stages, accompanied by peritoneal dissemination^46,47^. Despite the promising outcomes achieved through the administration of targeted therapies against aberrant DNA repair mechanisms, including PARPis^40,41^, HGSC eventually becomes resistant to therapy and worsens the prognosis of numerous patients^48^. Notably, the development of drug resistance in ovarian cancers also results in limited genetic alterations^49^, necessitating the implementation of alternative treatment strategies. Our results hold significant clinical potential, as similar anticancer effects of dietary modulations were observed on mice harboring PDXs derived from drug-resistant tumors. Wieder et al. ^5^ proposed that the UPR is a promising therapeutic target for various states of HGSC and that targeting the UPR along with a dietary intervention to promote stearate accumulation and limit the oleate content in tumors may constitute a novel therapeutic approach for refractory HGSC.

This study has some limitations. The detailed mechanisms by which stearate and palmitate exert different effects and the mechanisms by which oleate attenuates these effects remain unclear. Therefore, identifying a population for which the activation of the UPR with stearate is more effective is difficult. The effects on the immune system have not yet been investigated, and the details of their effects on normal organs are still unknown. Additionally, the dietary conditions employed here may lack direct applicability in clinical settings. Nonetheless, the implications of our study are noteworthy. Although dietary interventions are garnering increased attention in clinical research on cancer treatment^50^, they are generally regarded as complementary therapies. Our findings suggest that dietary modifications can exert direct antitumor effects, broadening the scope for dietary interventions in cancer treatment. These findings could be valuable in developing more solid evidence-based dietary interventions for cancer treatment.

## Supplementary information

Supplementary Information

Source data of Figure1-7

Source data of Supplementary FigureS1-S13

## Data Availability

The RNA-seq data from this study have been deposited in the Gene Expression Omnibus (GEO) under Accession No. GSE248408.
