# Supplementary material for: A stearate-rich diet and oleate restriction directly inhibit tumor growth via the unfolded protein response
Source: Exp Mol Med. 2024 Dec 2;56(12):2659–72. doi: 10.1038/s12276-024-01356-2 (PMC11671534; doi:10.1038/s12276-024-01356-2)
Supplement: Supplementary file 1 — Supplementary Information [file 12276_2024_1356_MOESM1_ESM.pdf]

Supplementary Figure 1

a

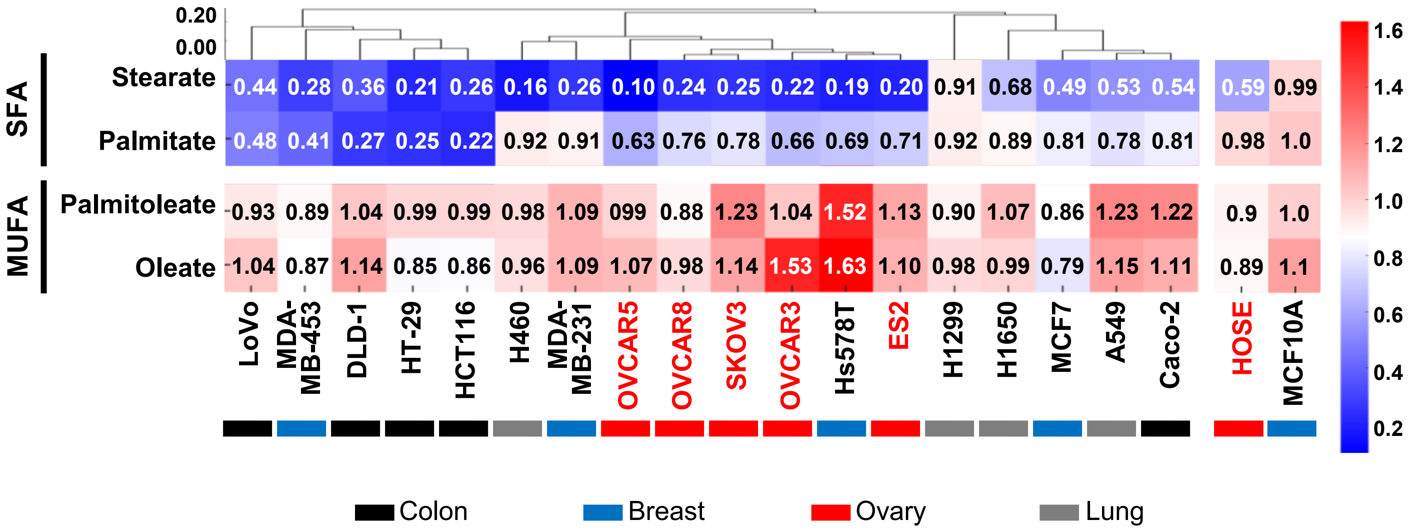

b

compound	IC_{50} Value ± SD (μM)				
	OVCAR5	OVCAR8	SKOV3	ES-2	OVCAR3
Stearate	36.96±3.22	31.04±1.97	37.19±0.46	59.89±6.32	90.94±16.37
Palmitate	1469.75±74.61	74.97±2.7	1230.93±258.69	1727.25±440.48	1781.50±26.11
Oleate	3145.5±1090.21	1009.93±175.96	1663.5±129.93	1691.25±363.65	2587.25±417.10

c

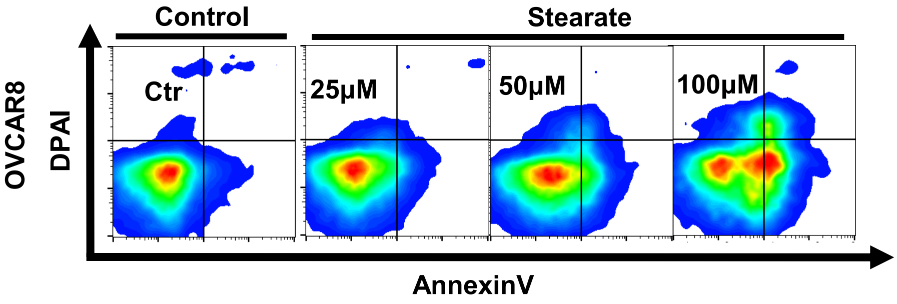

d

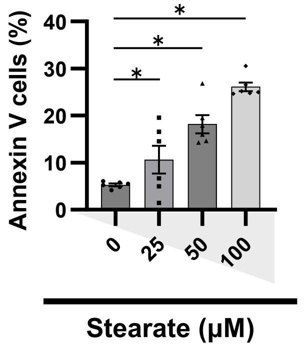

e

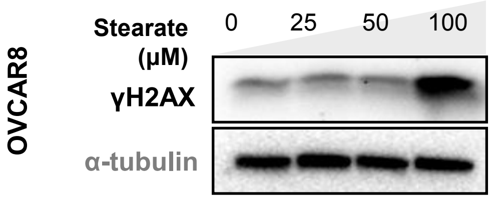

Supplementary Figure 2

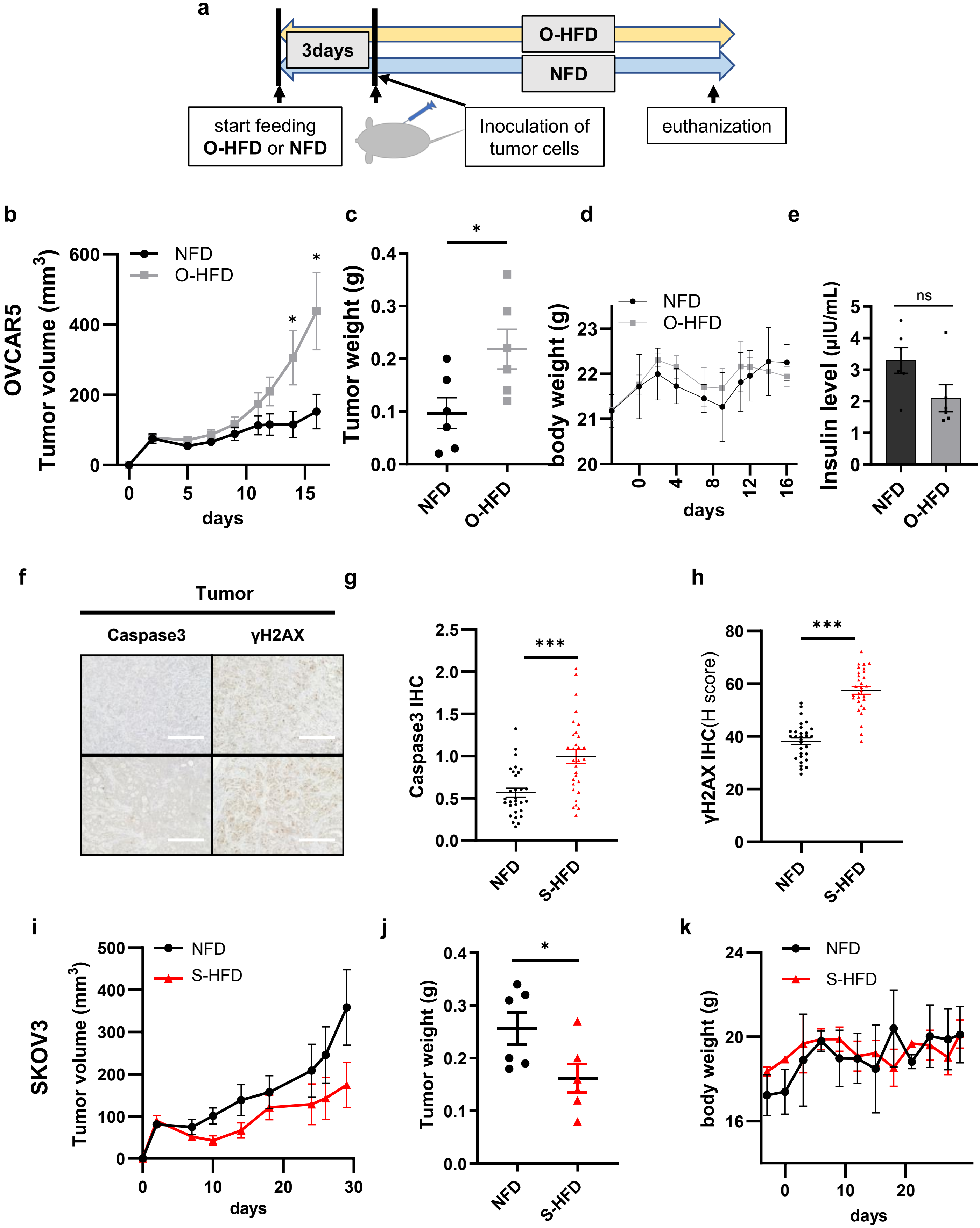

Supplementary Figure 3

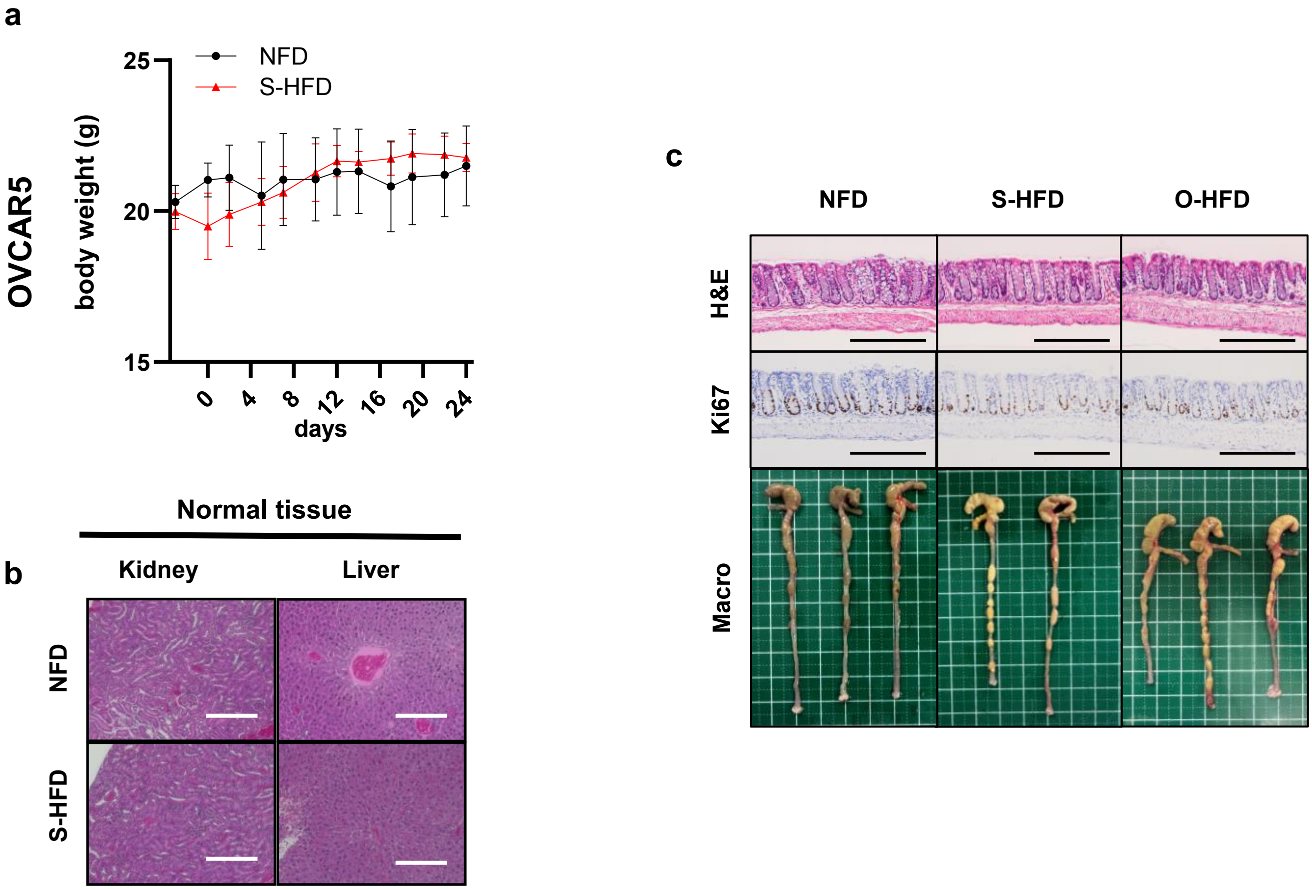

Supplementary Figure 4

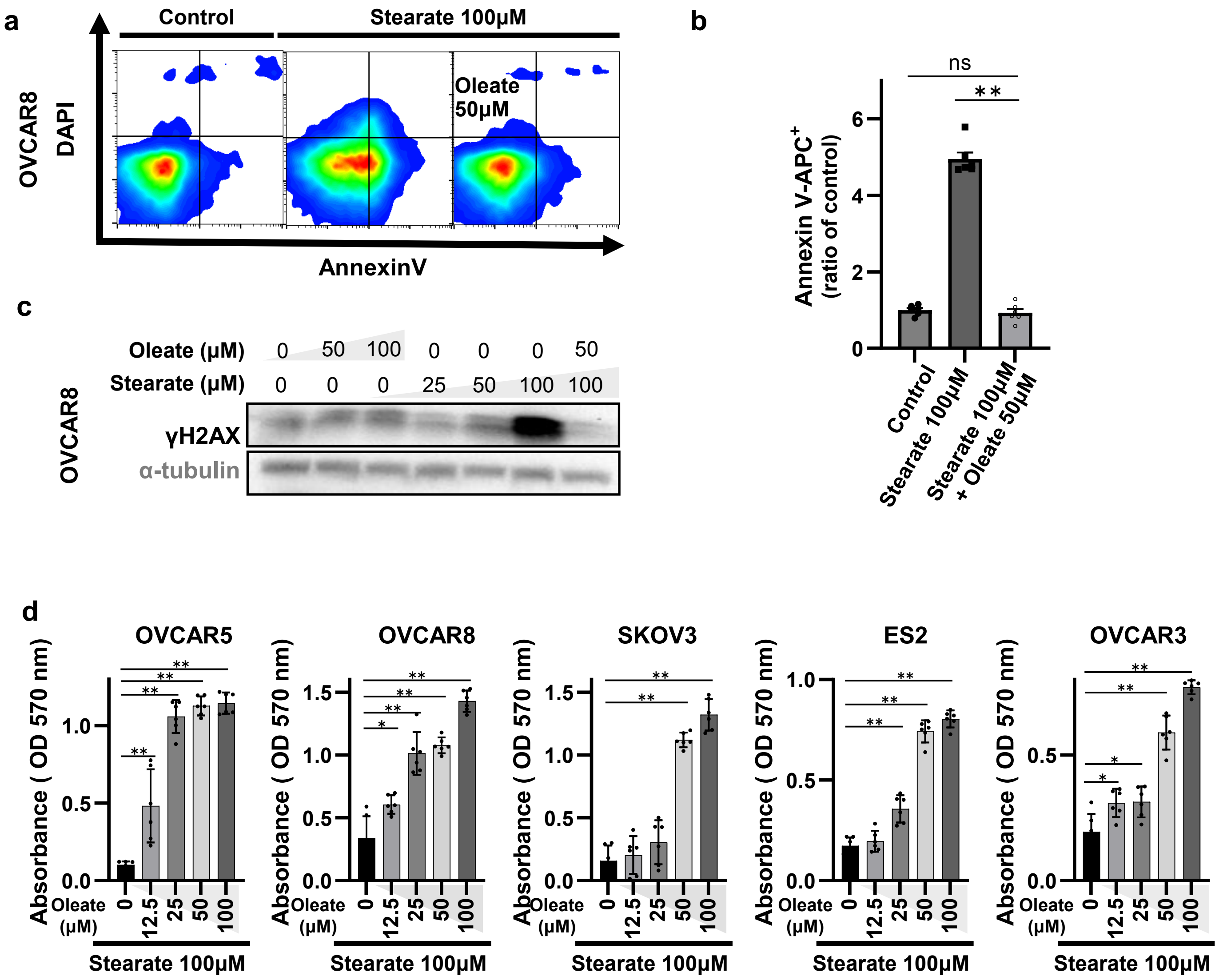

Supplementary Figure 5

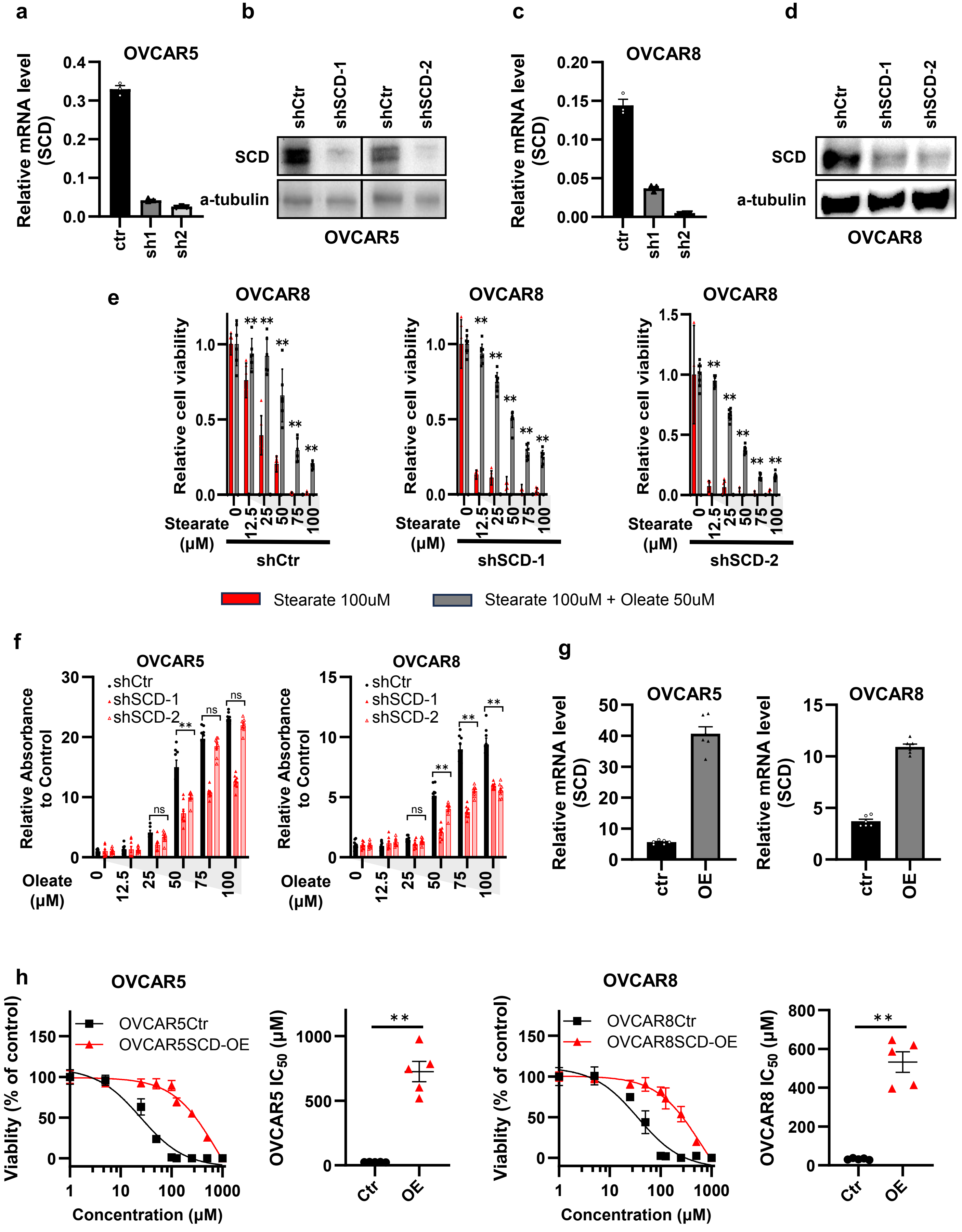

Supplementary Figure 6

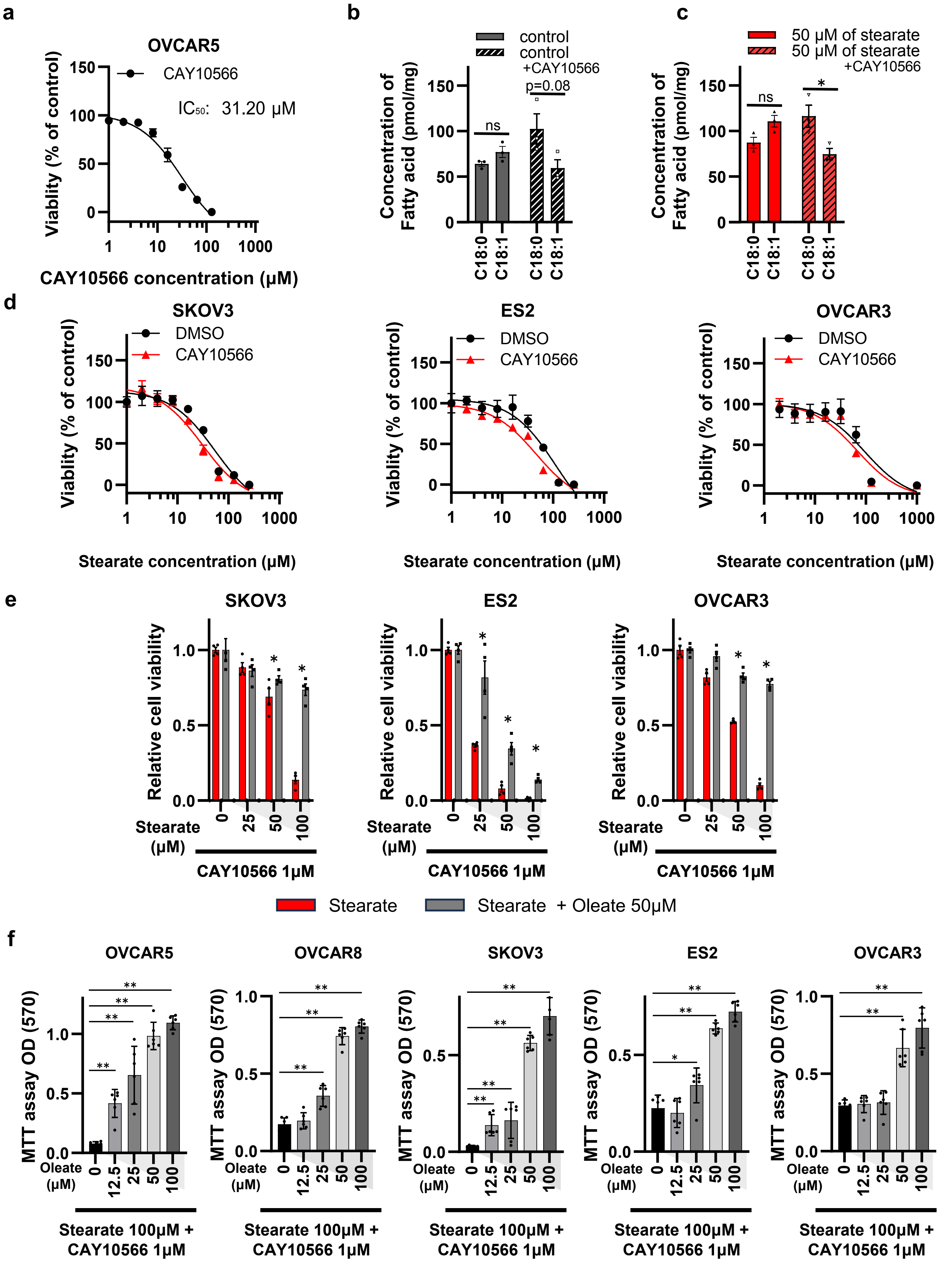

Supplementary Figure 7

a

Comparisons	UP	DOWN
Stearate vs Control	401	242
Stearate vs Oleate	192	598
Oleate vs Control	746	48

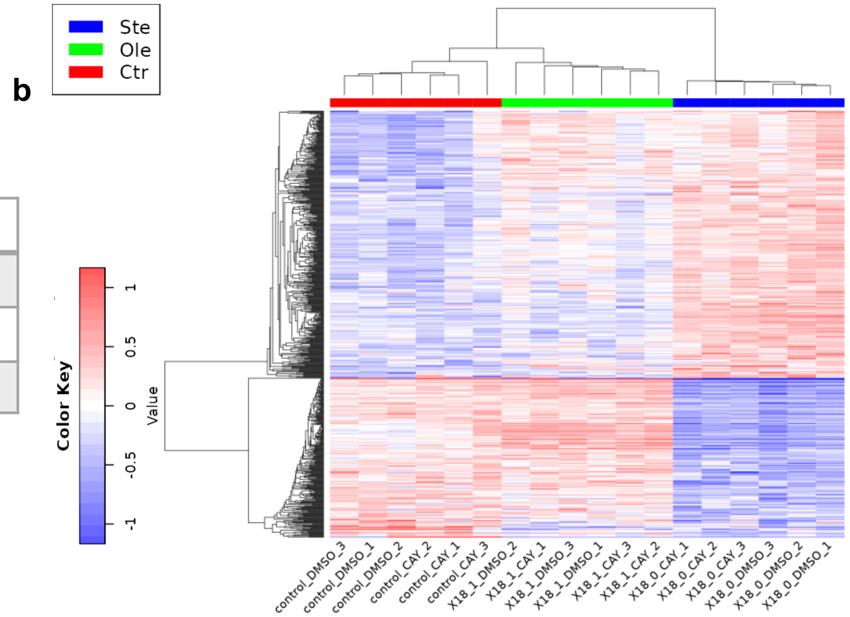

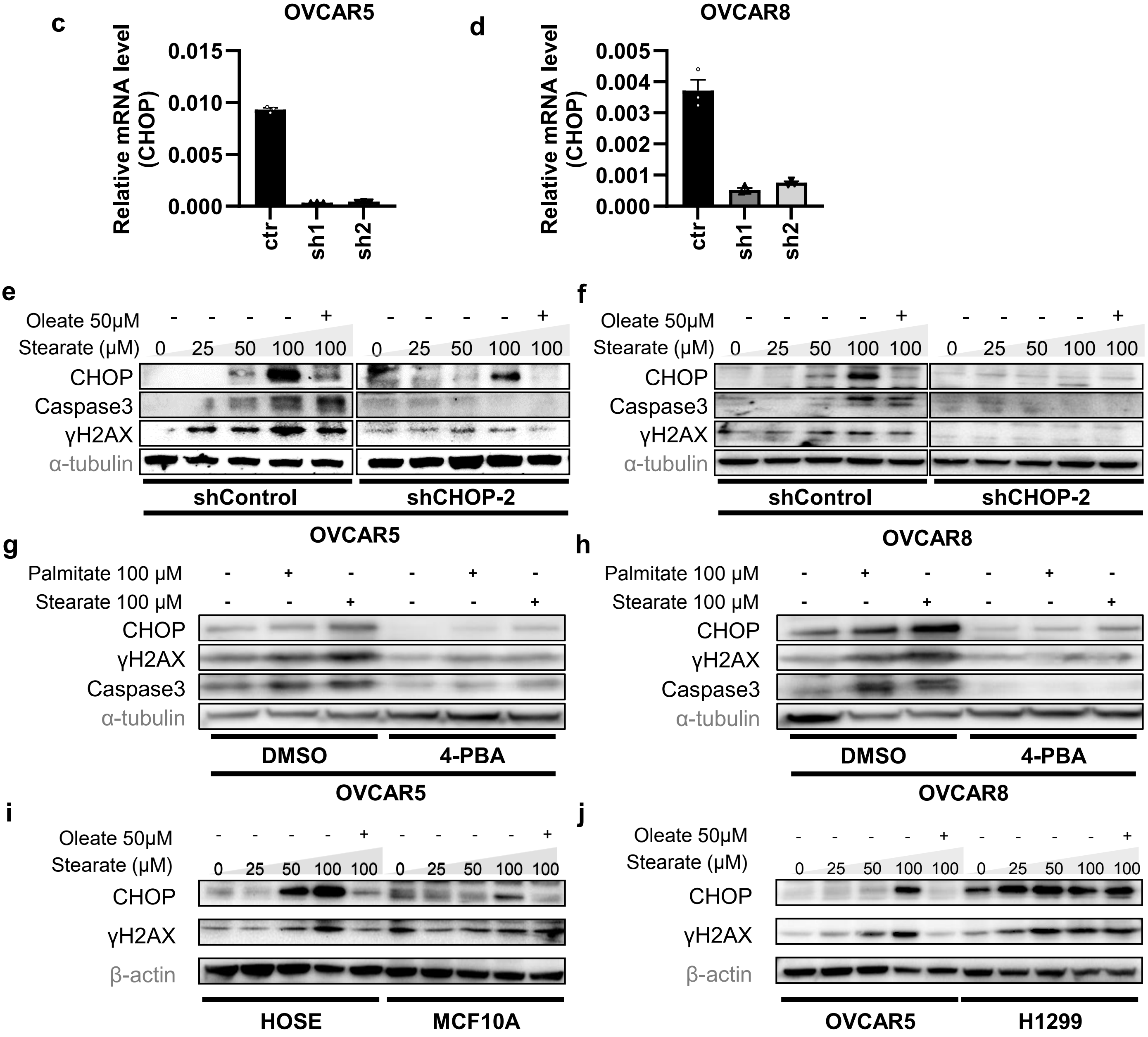

Supplementary Figure 8

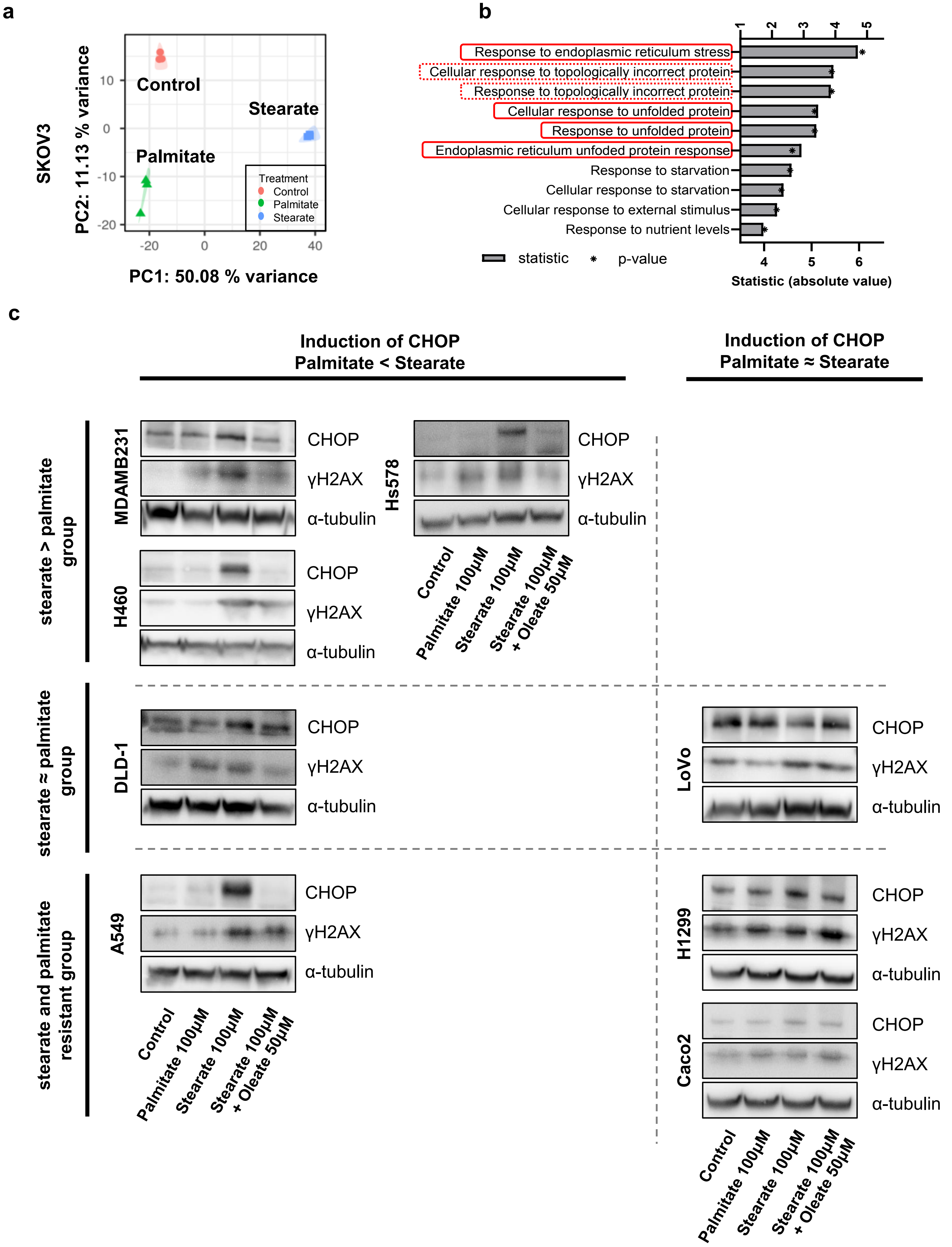

Supplementary Figure 9

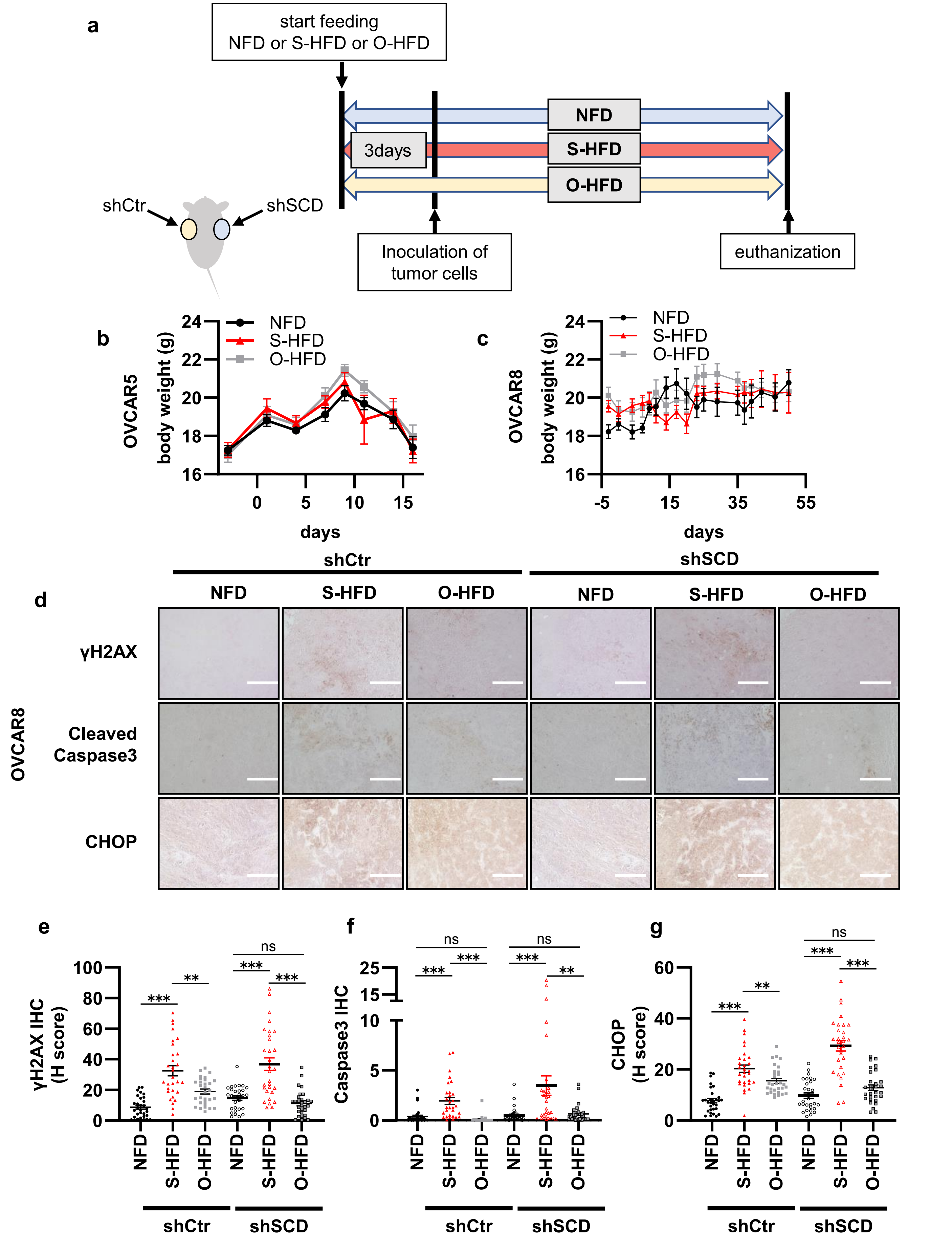

Supplementary Figure 10

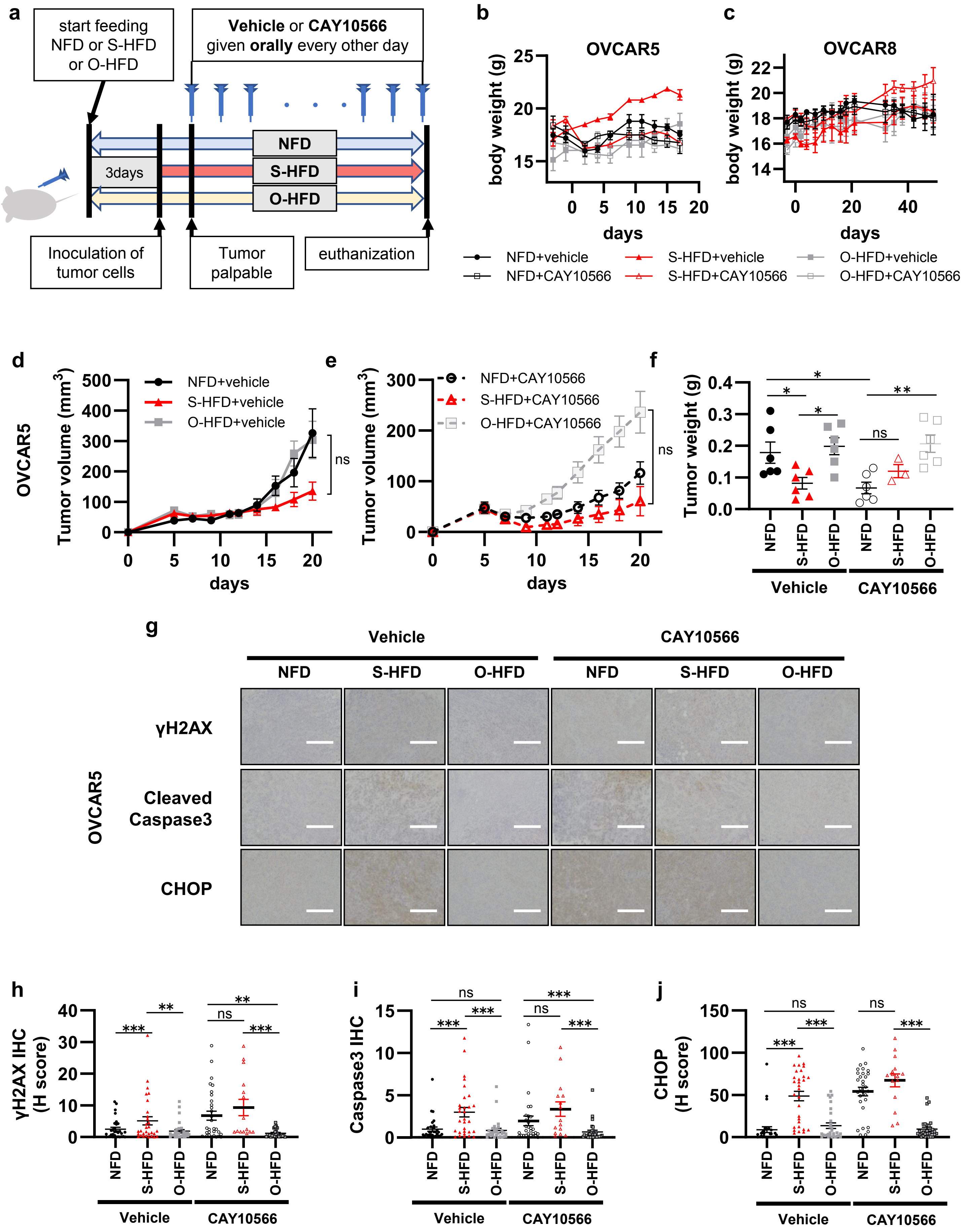

Supplementary Figure 11

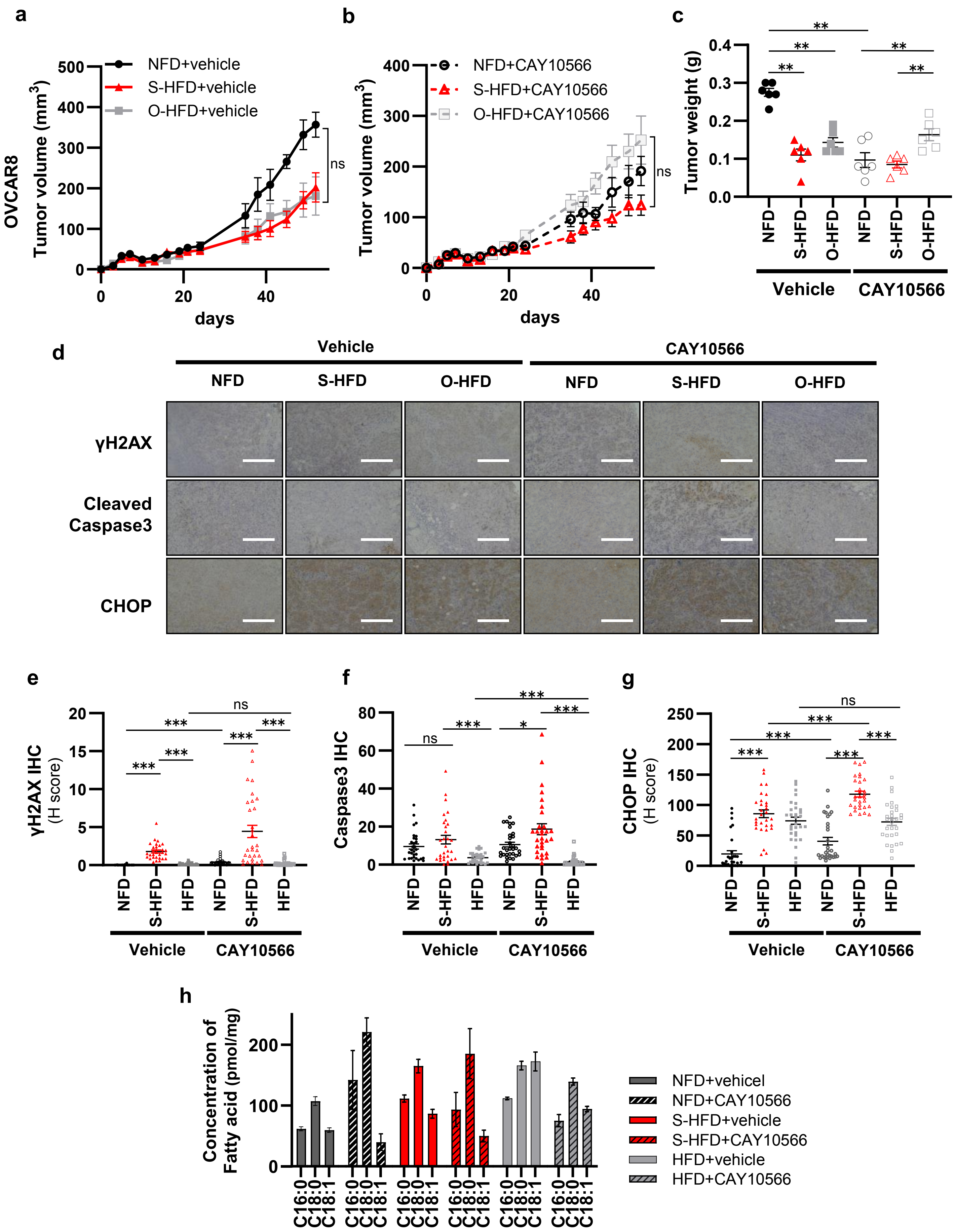

Supplementary Figure 12

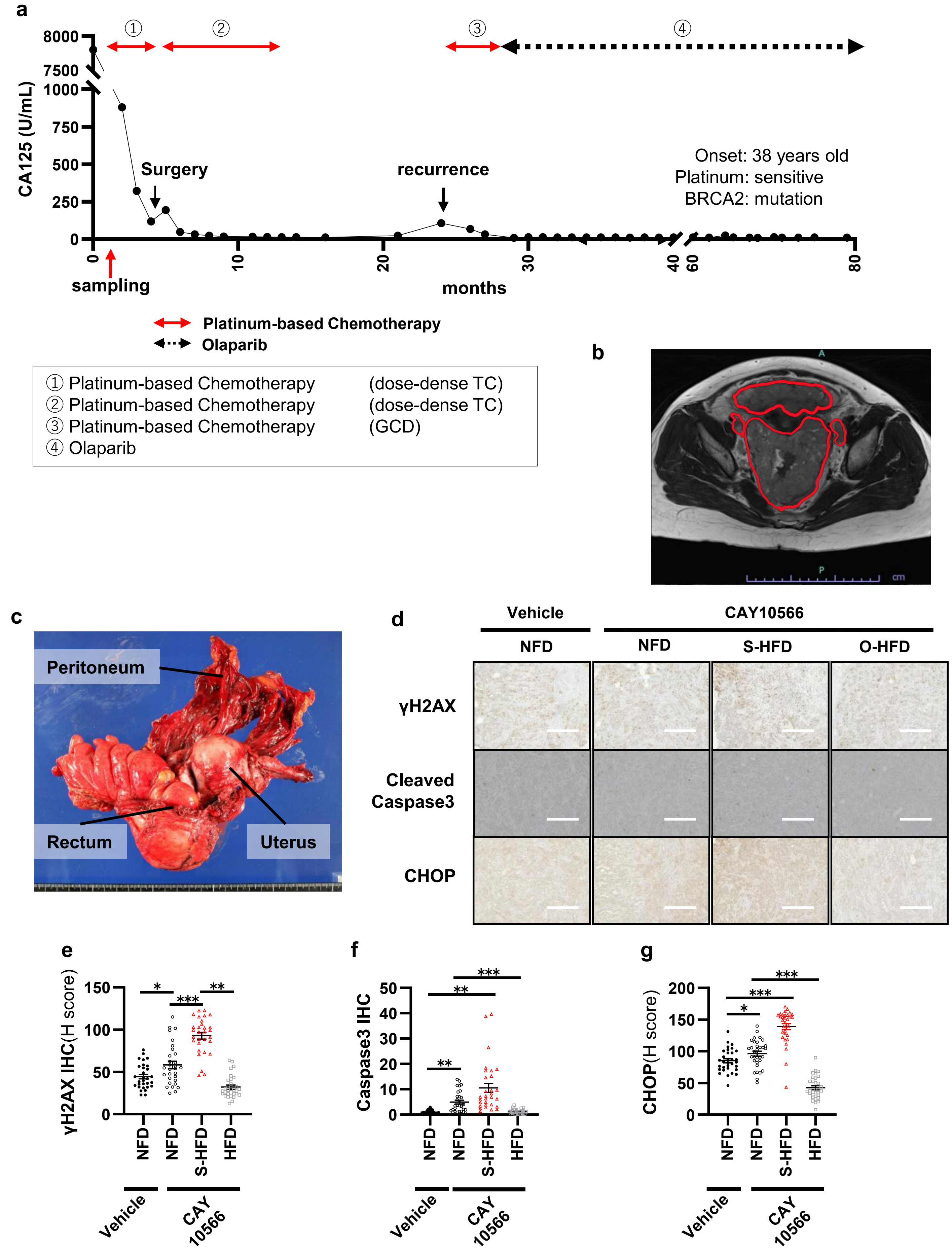

Supplementary Figure 13

a

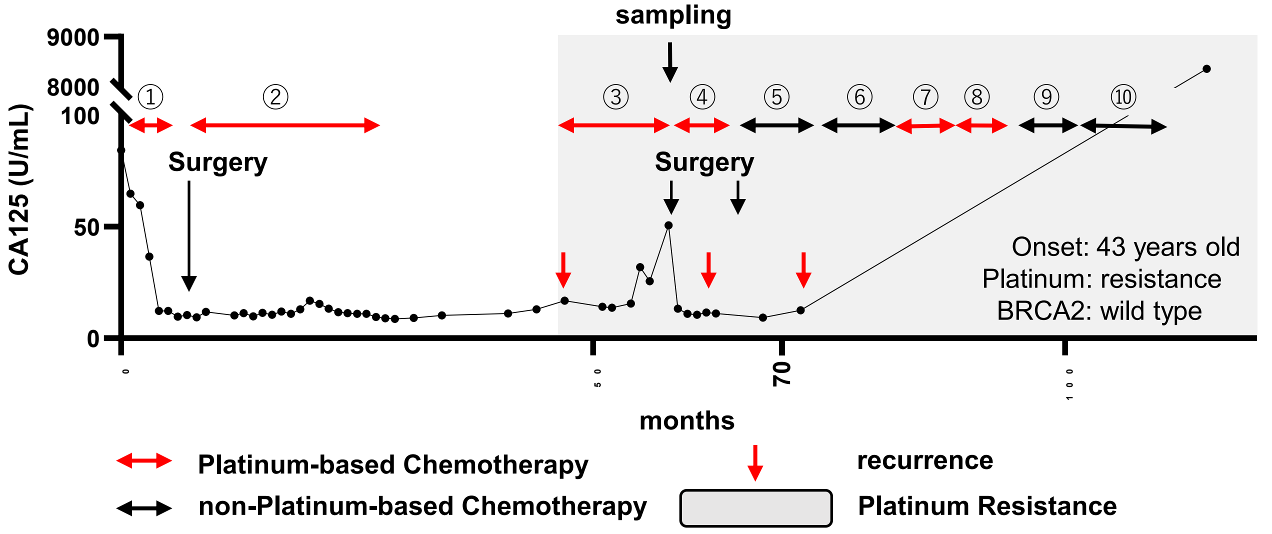

① Platinum-based Chemotherapy (TC)
② Platinum-based Chemotherapy (TC)
③ Platinum-based Chemotherapy (TC)
④ Platinum-based Chemotherapy (PLD/CBDCA)
⑤ non-Platinum-based Chemotherapy (GEM)
⑥ non-Platinum-based Chemotherapy (CPT-11)
⑦ Platinum-based Chemotherapy (VP)
⑧ Platinum-based Chemotherapy (NDP)
⑨ non-Platinum-based Chemotherapy (PTX+Bev)
⑩ non-Platinum-based Chemotherapy (NGT+Bev)

b

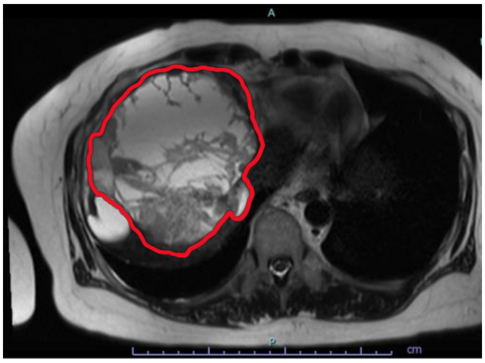

c

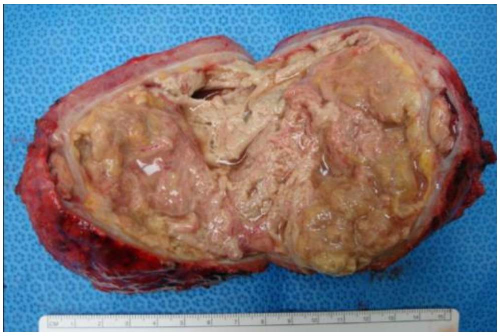

d

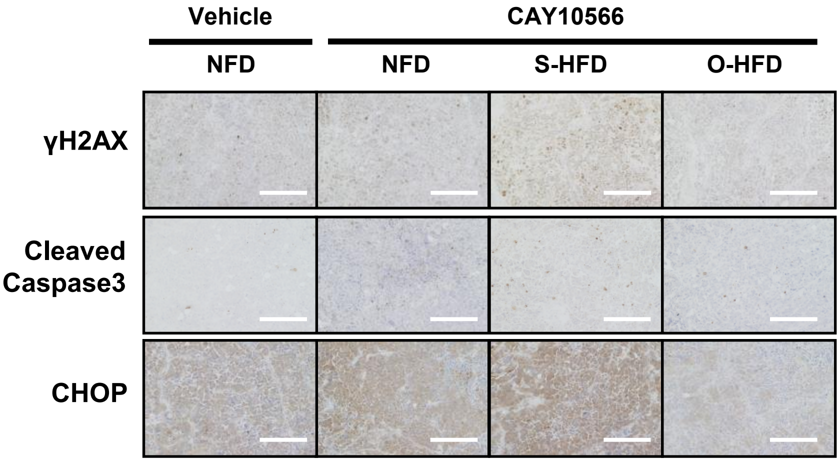

e

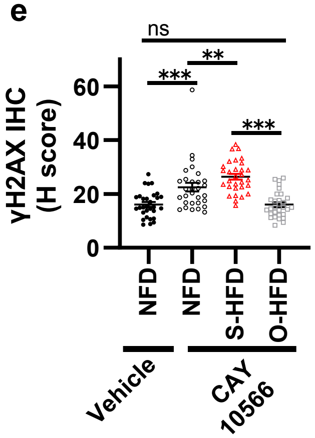

f

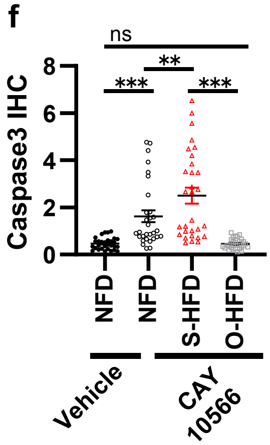

g

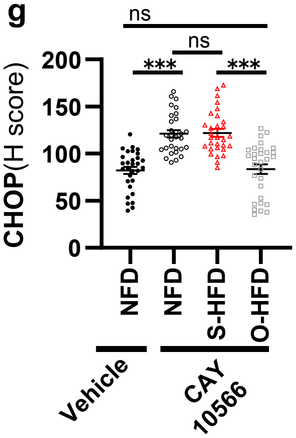

Supplementary Table 1. Compositions of Diets Used in This Study

		S-HFD (D12113001)		O-HFD (HFD32)		NFD (F-2)	
		% g	% kcal	% g	% kcal	% g	% kcal
Summary	Protein	26.0%	20.0%	25.5%	20.1%	20.8%	23.1%
	Carbohydrate	26.0%	20.0%	29.4%	23.2%	58.2%	64.7%
	Fat	35.0%	60.0%	31.9%	56.7%	4.8%	12.0%
	kcal/g	5.2		5.1		3.6	
		% g	%	% g	%	% g	%
Fat source	Soybean	3.2%	9.2%	-		4.8%	100.0%
	Safflower	-		20.0%	55.7%	-	
	Beef tallow(80% purity)	-		15.9%	44.3%	-	
	Cocoa butter	31.7%	90.5%	-		-	
Fatty acid composition (%)	C14:0	0.12%		1.10%		0.11%	
	C16:0	24.12%		12.60%		11.86%	
	C16:1(n-7)	0.24%		1.20%		0.14%	
	C18:0	33.35%		7.50%		4.74%	
	C18:1(n-9)	31.83%		64.30%		21.60%	
	C18:2(n-6)	7.83%		10.20%		52.58%	
	C18:3(n-3)	0.88%		0.20%		7.65%	

SUPPLEMENTARY FIGURE LEGENDS

Supplementary Fig. 1 Anti-proliferative effects of different fatty acids across various cancer cell lines and tumor-proliferative effects of an oleate-rich diet (O-HFD).

a. Proliferation assay and clustering: Data from a 72-h MTT assay across multiple cancer types and normal epithelial cells of the ovary (HOSE) and mammary gland (MCF10A) were subjected to k-means clustering. Cells were treated with various fatty acids (50 μM) and subjected to an MTT assay. Cell viability was normalized to that of a fatty acid-free control. Ovarian cancer cell lines are marked in red.

b. IC_{50} analysis data of Fig. 1c. IC_{50} values of stearate, palmitate, and oleate are shown, based on quadruplicate data. A 72 h MTT assay revealed a significantly reduced IC_{50} value for stearate across all tested cell lines.

c, d. Flow cytometry image of stearate-induced apoptosis with Annexin V/PI and bar graphs. Apoptosis in OVCAR8 ovarian cancer cells treated with varying stearate concentrations was analyzed using Annexin V/PI staining. Bar graphs (d) depict the percentage of Annexin V-positive cells compared to that in untreated controls, as determined using flow cytometry (c). Data represent mean ± SEM (n=4; *$p < 0.05$, Wilcoxon test).

e. γH2AX expression analysis using western blot. OVCAR8 cell lines were treated with the indicated stearate concentrations for 24 h and γH2AX expression was determined. α-tubulin served as the loading control.

Supplementary Fig. 2 Evaluating the in vivo effects of stearate-rich high-fat diet (S-HFD) or oleic acid-rich high-fat diet (O-HFD) using ovarian cancer cells.

a. Schematic diagram of an in vivo xenograft study. Specialized diets were initiated 3 days before cancer cell inoculation and maintained throughout the study.

b–e. Xenograft model using OVCAR5 cells. Mice were fed either a normal-fat diet (NFD) or a high-fat diet (O-HFD). (b) Tumor growth: Temporal curves illustrating xenograft tumor development. (c) Tumor weight: Quantitative analysis showing increased tumor mass in O-HFD mice upon harvest. (d) Body weight: No significant weight differences were observed between mice fed O-HFD or NFD. (e) Serum insulin levels: Serum insulin levels did not vary significantly between diet groups at study termination.

f. Immunohistochemistry of neoplastic tissues: Sections from NFD or S-HFD mice immunostained for cleaved caspase-3 and γH2AX. White scale bar, 200 μm.

g–h. Immunostaining quantification: Caspase-3 (g) and γH2AX (h) in panel (f) were upregulated in S-HFD mice. Cleaved caspase-3 and γH2AX were quantified by the stained area and IHC score, respectively (n=30; ***$p < 0.001$, Mann–Whitney test).

i–k. Result validation: Validation of the results shown in Fig. 2d, e using the SKOV3 cell line (n=6; *$p < 0.05$, Mann–Whitney test). Body weight analysis (k) showed no significant differences in body weight between S-HFD and NFD mice.

Supplementary Fig. 3 Effect of S-HFD and O-HFD on normal tissue in vivo.

a. Body weight analysis: Data from the cohort in Figs. 2d, e showed no significant differences in body weight between S-HFD and NFD mice.

b. H&E-stained tissues: Hematoxylin and eosin-stained renal and hepatic sections from mice on NFD or S-HFD. White scale bar, 200 μm.

c. Representative images of H&E staining, Ki67 staining, and gross appearance of the colon from control, S-HFD-fed, or O-HFD-fed mice for 25 days. Black scale bar, 200 μm.

Supplementary Fig. 4 Stearate induces apoptosis and DNA damage in ovarian cancer cells in vitro and in vivo while oleate mitigates stearate-induced cytotoxicity.

a, b. Flow cytometry image of stearate-induced apoptosis with Annexin V/PI and bar graphs. Apoptosis in OVCAR8 ovarian cancer cells treated with varying stearate concentrations was analyzed using Annexin V/PI staining. Bar graphs (b) depict the percentage of Annexin V-positive cells compared to that in untreated controls, as determined using flow cytometry (a). Data represent mean ± SEM (n=4; *$p < 0.05$, Wilcoxon test).

c. γH2AX expression analysis using western blotting. OVCAR8 cell lines were treated with the indicated stearate concentrations for 24 h and γH2AX expression was determined. α-tubulin served as the loading control.

d. Effects of oleate on 100 μM stearate-treated cells. OVCAR5, OVCAR8, SKOV3, ES-2, and OVCAR3 cells were treated with indicated oleate concentrations and co-incubated with 100 μM stearate for 72 h; viability was measured via an MTT assay (n=6; *$p < 0.05$, **$p < 0.01$, Mann–Whitney test).

Supplementary Fig. 5 Dose-dependent mitigation effect of exogenous oleate on stearate-induced cytotoxicity.

a, c. SCD knockdown and RT-qPCR validation: shRNAs (shSCD-1 and shSCD-2) were used to knock down SCD in OVCAR5 and OVCAR8 cells, and results were confirmed using RT-qPCR.

b, d. Western blot analysis of SCD protein following shSCD-1 and shSCD-2-mediated knockdown in OVCAR5 (b) and OVCAR8 cells (d).

e. Effects of oleate on stearate-treated shSCD-transfected OVCAR8 cell viability. Validation of the results shown in Fig. 3d. Cells were treated with varying concentrations of stearate for 72 h; viability was measured via an MTT assay (n=6; *$p < 0.05$, **$p < 0.01$, Mann–Whitney test).

f. MTT assay of shSCD-transfected cells: OVCAR5 and OVCAR8 cells transfected with shSCD were cultured with 100 µM stearate and treated with varying concentrations of oleate for 72 h. Data were normalized to the condition with 0 µM stearate concentration. Oleate led to a concentration-dependent reversal of stearate-induced cell death (n=8; ns: no significance, **$p < 0.01$, Mann–Whitney test).

g. SCD overexpression and RT-qPCR validation: Open reading frames were used to overexpress SCD in OVCAR5 and OVCAR8 cells, and results were confirmed using RT-qPCR.

h. IC_{50} values of stearate in SCD-overexpressed OVCAR5 cells and OVCAR8 cells. SCD overexpression significantly attenuated the toxicity of stearate (n=6; $^{**}p < 0.01$, Mann–Whitney test.

Supplementary Fig. 6 Low-dose CAY10566 decreases oleate concentrations and enhances stearate-induced cytotoxicity.

a. IC_{50} values of CAY10566 in OVCAR5 cells.

b, c. LC/MS fatty acid quantification in OVCAR5 cells cultured under various conditions (n=3; $^{*}p < 0.05$, $^{**}p < 0.01$, n: no significance, unpaired t-test).

d. IC_{50} of stearate with 1 μM CAY10566 and DMSO control. Validation of the results shown in Figs. 3e, f using SKOV3, ES-2, and OVCAR3 cells, showing a significant reduction. Data are from five independent experiments.

e. Cell viability in response to treatment with stearate and oleate under 1 μM CAY10566. Validation of the results shown in Fig. 3e (n=4; $^{*}p < 0.05$, Mann–Whitney test).

f. Effects of oleate on the viability of 100 μM stearate- and 1 μM CAY10566-treated cells. OVCAR5, OVCAR8, SKOV3, ES-2, and OVCAR3 cells were treated with indicated oleate concentrations and co-incubated with 100 μM stearate and 1 μM CAY10566 for 72 h; viability was measured via an MTT assay (n=6; $^{*}p < 0.05$, $^{**}p < 0.01$, Mann–Whitney test).

Supplementary Fig. 7 Contrasting effects of stearate and oleate on cells in terms of the CHOP activation–DNA damage–apoptosis axis.

a. Gene expression in OVCAR5 cells: Differential gene expression in cells treated with 50 μM stearate or oleate or untreated cells (FDR < 0.05, fold change > 1.25).

b. Heatmap showcases pathways involving 643 genes differentially expressed between stearate-treated and control OVCAR5 cells.

c, d. CHOP knockdown and RT-qPCR validation: shRNAs (shCHOP-1 and shCHOP-2) were used to knock down CHOP in OVCAR5 cells (c) and OVCAR8 cells (d), and results were confirmed using RT-qPCR.

e, f. Result validation of western blot analysis: Validation of the results shown in Figs. 4e, f using shCHOP-2-transfected OVCAR5 cells (e) and OVCAR8 cells (f). α-tubulin was used as an internal control.

g, h. Representative western blot analysis of protein expression of CHOP, cleaved caspase 3, and γ-H2AX treated with 4-PBA along with 100 μM of palmitate or stearate in OVCAR5 cells (g) and OVCAR8 cells (h). α-tubulin was used as an internal control.

i, j. Representative western blot analysis of proteins involved in the unfolded protein response (UPR) and DNA damage in HOSE and MCF10A cells (i) and OVCAR5 and H1299 cells (j) treated with stearate and oleate at the indicated concentrations. β-actin was used as an internal control.

Supplementary Fig. 8 Differential sensitivity to stearate and palmitate across cell lines.

a. RNA-Seq in SKOV3 cells subjected to various treatments. Cells were treated and cultured for 24 h before RNA-Seq. Principal component analysis revealed distinct gene expression profiles without treatment-based separation.

b. Top 10 functionally enriched GO pathways. Biological processes induced by stearate compared with those induced by palmitate are shown. Pathways related to UPR are circled in red, and pathways associated to incorrect protein are circled in red dotted lines.

c. Representative western blot analysis of γH2AX and CHOP in various cancer cell lines derived from different tissues. These cell lines are shown based on differences in their sensitivity to fatty acids: stearate > palmitate, stearate ≈ palmitate, and stearate- and palmitate-resistant groups. α-tubulin was used as an internal control.

Supplementary Fig. 9 Evaluating the in vivo effects of SCD-KD with S-HFD or O-HFD using OVCAR8 cells.

a. Experimental schematic for in vivo experiments involving SCD knockdown. Control shRNA (shCrt) cells were subcutaneously injected into the left flank of the mouse, while SCD-knockdown (shSCD) cells were injected into the right flank.

b, c. Body weight data of the cohort in Figs. 5a–c and Figs. 5d, e. Mean ± SEM of body weights in mice subjected to various diet and treatment conditions (n=6; no significance detected).

d. Representative immunohistochemical images of tumor tissues derived from shCtr-OVCAR8 cells or shSCD-1-OVCAR8 cells depicting the expression of caspase-3, γH2AX, and CHOP.

e–g. Quantitative analysis of caspase-3, γH2AX, and CHOP expression in tissues (n=30; $^{**}p < 0.01$, $^{***}p < 0.001$, ns: no significance, Mann–Whitney test).

Supplementary Fig. 10 Evaluating the in vivo effects of SCD-inhibitor (CAY10566) with S-HFD or O-HFD using OVCAR5 cells.

a. In vivo study design involving CAY10566. Vehicle or CAY10566 was administered via oral gavage bi-daily after tumor detection.

b, c. Body weight data of the cohort in Supplementary Figs. S9d–f and Supplementary Figs. S10a–c: Mean ± SEM of body weights in mice subjected to various diet and treatment conditions.

d, e. Tumor growth in mice: Tumor size was monitored following subcutaneous injection of OVCAR5 cells in mice fed NFD, S-HFD, or O-HFD, with either vehicle or CAY10566 treatment. Data are presented as the mean ± SEM (n=6; ns: no significance, Mann–Whitney test).

f. Weight of the tumors at the end of the experimental period (n=6; *p < 0.05, **p < 0.01, ns: no significance, Mann–Whitney test).

g. Representative immunohistochemical images of tumor tissues derived from OVCAR5 cells following indicated treatment, depicting the expression of caspase-3, γH2AX, and CHOP.

h–j. Quantitative analysis of caspase-3, γH2AX, and CHOP expression in tissues (n=30; **p < 0.01, ***p < 0.001, ns: no significance, Mann–Whitney test).

Supplementary Fig. 11 Validation of the in vivo effects of SCD-inhibitor (CAY10566) with S-HFD or O-HFD via regulation of stearate and oleate concentration using OVCAR8 cells.

a, b. Tumor growth in mice: Tumor size was monitored following subcutaneous injection of OVCAR8 cells in mice fed NFD, S-HFD, or O-HFD, with either vehicle or CAY10566 treatment. Data are presented as the mean ± SEM (n=6; ns: no significance, Mann–Whitney test).

c. Weight of the tumors at the end of the experimental period (n=6; **p < 0.01, Mann–Whitney test).

d. Representative immunohistochemical images of tumor tissues derived from OVCAR8 cells with indicated treatment depicting the expression of caspase-3, γH2AX, and CHOP.

e–g. Quantitative analysis of caspase-3, γH2AX, and CHOP expression in tissues (n=30; *$p < 0.05$, ***$p < 0.001$, ns: no significance, Mann–Whitney test).

h. Tumor fatty acid profiles: LC/MS analysis of C18:1 and C18:0 in tumors resected from mice of the same cohort in Supplementary Figs. S9a, d–f subjected to different diet and treatment conditions.

Supplementary Fig. 12 Clinical course of the patient for PDX82 and confirmation of activation of the UPR and apoptosis in vivo.

a. Longitudinal CA-125 levels tracked alongside therapeutic interventions in the patient for PDX82, as depicted in Fig. 6a.

b. MRI of the intra-abdominal neoplasm. T2-weighted MRI of the pelvic region highlighting the tumor location.

c. Surgical sample from interval debulking surgery.

d. Representative immunohistochemical images of PDX82 tumors. Expression levels of caspase-3, γH2AX, and CHOP in PDX82 tumors resected from mice subjected to various diets and treatments.

e–g. Quantitative analysis of caspase-3, γH2AX, and CHOP expression in tissues (n=30; *$p < 0.05$, **$p < 0.01$, ***$p < 0.001$, Mann–Whitney test).

Supplementary Fig. 13 Clinical course of the patient for PDX72 and confirmation of activation of the UPR and apoptosis in vivo.

a. Longitudinal CA-125 levels tracked alongside therapeutic interventions in the patient for PDX72, as depicted in Fig. 6g.

b. MRI of the intra-abdominal neoplasm. T2-weighted MRI of the hepatic region highlighting the tumor location.

c. Surgical sample from surgery.

d. Representative immunohistochemical images of PDX72 tumors. Expression levels of caspase-3, γH2AX, and CHOP in PDX72 tumors resected from mice subjected to various diets and treatments.

e–g. Quantitative analysis of caspase-3, γH2AX, and CHOP expression in tissues (n=30; $**p < 0.01$, $***p < 0.001$, ns: no significance, Mann–Whitney test).

SUPPLEMENTARY MATERIALS AND METHODS

Cell culture

All cells were cultured at 37 °C in an incubator supplied with 5% CO_2. All cell lines were routinely screened for mycoplasma contamination, with the last test conducted on January 22, 2024, using a Mycoplasma PCR Detection Kit. All cell line-based experiments were performed prior to the 10th passage.

Reagents

For in vitro experiments, CAY10566 was suspended in dimethyl sulfoxide (DMSO) to prepare a 1-mM stock solution. For in vivo application, CAY10566 was formulated in methylcellulose at 0.5 mg/mL and administered via oral gavage at a dose of 2.5 mg/kg every 12 h. Methylcellulose was used as the vehicle. 4-Phenylbutyric acid (4-PBA) was dissolved in DMSO to create a 5 mM stock solution, which was stored at -30 °C until use. For in vitro cell culture studies, 4-PBA was added to the culture medium to achieve a final concentration of 5 μM.

Fatty acid preparation

A 50 mM free fatty acid (FFA) sodium solution was prepared by dissolving the fatty acids in 50 mM sodium hydroxide (NaOH) and heating the mixture between 70 and 80 °C in a gently shaking water bath. This solution was then combined with FFA-free bovine serum albumin (FFA-free BSA) preheated to 55 °C. The mixture was agitated vigorously for 10 s and subsequently incubated at 55 °C for an additional 15 min in a shaking water bath to facilitate conjugation. The conjugated FFA-BSA solution was cooled to 25 °C, passed through a 0.45 μm-pore-size membrane filter, and stored at −20 °C until subsequent experimental use.

Cell viability assay

Cells were seeded (7.5×10^3 cells/well) in 96-well plates and treated with 50 μM of either palmitate, stearate, palmitoleate, or oleate for 72 h. To assess cell viability, 100 μL of a 500 μg/mL 3-[4,5-dimethylthiazol-2-yl]-2,5 diphenyl tetrazolium bromide solution (MTT) prepared in RPMI-1640 medium was added to each well. The plates were incubated at 37 °C in an incubator for 2 h, after which the MTT solution was aspirated, and the formed formazan crystals were solubilized in 100 μL methanol. Cell viability was quantified by measuring the absorbance at 570 nm using an iMark Microplate Reader. Cell viability under each experimental condition was analyzed using four to eight replicates.

Proliferation assay

Cells were seeded (7.5×10^3 cells/well) in 96-well plates and treated with either FFAs or CAY10566 at the indicated concentrations. At 24, 48, and 72 h post-incubation, the culture medium was removed, the cells were washed once with phosphate-buffered saline (PBS), and cell viability was assessed using the MTT assay.

Stable knockdown using shRNAs
Stable OVCAR5 and OVCAR8 knockdown cell lines were generated via lentiviral transfection using two distinct lentiviral shRNAs targeting SCD and CHOP and a non-silencing control. For SCD, sh1 (Catalog# TRCN0000327814) and sh2 (Catalog# TRC0000327815) were purchased from Sigma-Aldrich; for CHOP, sh1 (Catalog# pDNA (VB900039-3848rmh)), sh2 (Catalog# pDNA (VB900039-3854gdh)), and short (Catalog# VB010000-0009mxc) were obtained from Vector Builder. After transfection, the cells were selected using puromycin, and the efficacy of knockdown was verified using RT-qPCR and western blot analyses.

Gene overexpression using lentiviral vectors
A lentiviral vector encoding the SCD gene was transfected into the OVCAR5 and OVCAR8 cells to construct SCD-overexpressing cell lines, termed OVCAR5-OE-SCD and OVCAR8-OE-SCD, respectively. An empty lentiviral vector was used as a control. The transfection of cells with lentivirus was conducted according to the manufacturer's instructions.

The SCD overexpression vector (pLV[Exp]-EGFP：

T2A:Puro-EF1A>hSCD[NM_005063.5] (Vector ID: VB900005-7925zug)) and control GFPexpressing vector, Vector ID; VB010000-9298rtg were obtained from VectorBuilder.

IC_{50} determination
Cells were seeded (7.5×10^3 cells/well) into 96-well plates, and their viability in response to FFAs or CAY10566 was assessed after 72 h treatment. For the combination treatments, stearate, CAY10566, and oleate were added simultaneously at the specified concentrations, and cell viability was evaluated using the MTT assay.

Flow cytometry
Apoptosis was quantified using the Annexin V APC Apoptosis Assay Kit and flow cytometry. For in vitro experiments, cells cultured in 60 mm dishes were harvested after 24 h treatment with FFAs at the designated concentrations. For in vivo experiments, tumors from mice fed either an NFD or S-HFD were dissociated into single-cell suspensions using a gentleMACS Octo Dissociator with Heater and a Tumor Dissociation Kit for humans, according to the manufacturer's protocol.

Enzyme-linked immunosorbent assay (ELISA)
Blood samples were collected from mice via cardiac puncture during euthanasia. Serum insulin levels were quantified using a Mouse Insulin ELISA Kit. All standards and samples were analyzed in sextuplicate.

Western blot analysis
The cells were lysed in a buffer containing triethanolamine-HCl supplemented with 0.2% sodium dodecyl sulfate, 1% Triton X-100, and a complete-mini protease inhibitor cocktail. Cell lysates were sonicated for 15 min, and protein concentrations were determined using the Pierce-BCA protein assay kit with BSA as the standard. Equal

amounts of protein were subjected to electrophoresis on a bis-tris gradient polyacrylamide gel and subsequently transferred onto polyvinylidene fluoride membranes. The membranes were blocked for 1 h at room temperature using Blocking One. They were then incubated overnight at 4 °C with primary antibodies under gentle shaking. Following washing with Tris-Buffered Saline with 0.1% Tween 20, the membranes were incubated with horseradish peroxidase (HRP)-conjugated secondary antibodies for 1 h at room temperature.
All experiments were performed in triplicate.
Immunoreactive signals were detected using the WesternBright ECL kit and the ChemiDoc™ XRS+ system and quantified using Image Lab 2.0 software.

IHC and immunofluorescence
IHC was performed using the streptavidin-biotin peroxidase complex method as previously reported[1]. Epitope retrieval was performed at 90 °C for 20 min in 0.01 M of citrate buffer (pH 6.0), mixing 0.01 M of citric acid monohydrate and 0.01 M of trisodium citrate dihydrate. Tumor samples were incubated with specific primary antibodies overnight at 4 °C, followed by a 1-h incubation with biotinylated goat anti-rabbit secondary antibodies at room temperature. IHC images were captured using a BZ-9000 microscope and processed for H and DAB channels using the Color Deconvolution2 plugin in ImageJ software. The threshold was adjusted for each experiment, and H scores were calculated by multiplying the intensity score by the area percentage for each population present in a given tumor sample: H Score = 1 × (% of 1+areas) + 2 × (% of 2+areas) + 3 × (% of 3+areas).

Reverse-transcription PCR and real-time quantitative PCR
Total RNA was isolated using an RNeasy Mini Kit and reverse-transcribed using the ReverTra Ace qPCR RT Kit according to the manufacturer's guidelines. Gene amplification was conducted using the PowerUp SYBR Green Master Mix on a StepOnePlus Real-Time PCR System. Relative mRNA expression levels were ascertained using the ΔΔCt method, with SCD expression normalized to β-actin expression and CHOP expression normalized to GAPDH expression.

Liquid chromatography with tandem mass spectrometry (LC-MS/MS)
For in vitro experiments, 2×10^6 OVCAR5 cells were seeded onto six 10-cm dishes and cultured for 24 h. Next, the plates were individually cultured for an additional 24 h in the following media:
(1) medium containing 5% FFA-free BSA with DMSO as the control vehicle
(2) medium containing 5% FFA-free BSA with 1 µM CAY10566
(3) medium containing 5% FFA-free BSA with 50 µM stearate and DMSO
(4) medium containing 5% FFA-free BSA with 50 µM stearate and 1 µM CAY10566
(5) medium containing 5% FFA-free BSA with 50 µM oleate and DMSO
(6) medium containing 5% FFA-free BSA with 50 µM oleate and 1 µM CAY10566.
The cells were then washed twice with ice-cold PBS, harvested using a disposable scraper in 700 µL ice-cold saline, transferred to 1.5-mL collection tubes, and centrifuged at 100 ×g at 4 °C for 15 min. After aspirating the supernatants, the cell pellets were weighed, snap-frozen in liquid nitrogen, and stored at −80 °C.

For in vivo experiments, tumors harvested from mice harboring OVCAR5 xenografts subjected to CAY10566 treatment were used. The tumors were sliced into approximately 2–3 mm^3 cubes, transferred to 1.5-mL collection tubes, weighed, snap-frozen in liquid nitrogen, and stored at −80 °C.
For in vitro and in vivo experiments, pre-weighed, dimple-processed polypropylene tubes, supplied as part of a Biomasher II, were used. Ethanol containing oleate-d9 at 0.5 pmol/μL as an internal standard (IS) was added to the collection tubes containing cells or tumors at 0.1 mg/μL. The samples were homogenized using dimple-processed polyoxymethylene stir bars, which were also part of the Biomasher II. The homogenates were vortexed for 30 min at 4 °C and centrifuged at 16000 ×g for 10 min at 4 °C. The supernatants were transferred to new 1.5-mL polypropylene tubes and stored at −80 °C until further analysis.
For analysis, the stored samples were diluted 1:4 with ethanol and transferred to polymethylpentene vials, and 3 μL samples were injected into the LC-MS/MS system. An isocratic gradient of A99%/B1% at 0.4 mL/min for 9.01 min was used for separation; ethanol and 90% 2-propanol/10% acetonitrile/10 mM ammonium formate/0.1% formic acid were used as solvents A and B, respectively. ACQUITY UPLC BEH C18 1.7 μm, 2.1 × 100 mm set to 40 °C was used as the separation column. Simultaneous analyte detection and analyses were performed using pseudo-multiple reaction monitoring (pMRM) combined with negative-polarity electrospray ionization. The following pMRM transitions were used: oleate (281.05 > 281.25 m/z, CE 12 V), palmitate (255.05 > 255.25 m/z, CE 12 V), stearate (283.15 > 283.25 m/z, CE 14 V), and oleate-d9 (290.20 > 290.30 m/z, CE 13 V). Peaks were analyzed using LabSolutions software (version 5.99, Shimadzu). The abundance of each analyte was calculated by normalizing the peak areas of the analytes to those of the IS, followed by application to the corresponding calibration curves for each analyte. Calibration curves were generated by processing samples prepared from untreated OVCAR5 cell homogenates spiked with varying amounts of analytes. All plastic tubes, stir bars, and vials were washed with ethanol and dried prior to use.

RNA sequencing
OVCAR5 cells (3×10^5) were seeded into six-well plates and cultured for 24 h under standard conditions. Following the initial 24 h culture period, the cells were subjected to different treatment conditions for an additional 24 h in the following media:
(1) medium containing 5% FFA-free BSA with DMSO as the control vehicle
(2) medium containing 5% FFA-free BSA with 1 μM CAY10566
(3) medium containing 5% FFA-free BSA with 50 μM stearate and DMSO
(4) medium containing 5% FFA-free BSA with 50 μM stearate and 1 μM CAY10566
(5) medium containing 5% FFA-free BSA with 50 μM oleate and DMSO
(6) medium containing 5% FFA-free BSA with 50 μM oleate and 1 μM CAY10566.
Each treatment was conducted in triplicate to ensure the reproducibility and reliability of the data. Following the 24 h treatment period, the cells were harvested for RNA sequencing analysis. Total RNA from cells was extracted using an RNeasy Micro kit according to the supplier's instructions.
In a subsequent RNA sequencing experiment, OVCAR5, OVCAR8, and SKOV3 cells were similarly seeded into six-well plates (3×10^5 cells per well) and cultured for 24 h

under standard conditions. The cells were then exposed to the following treatment conditions for an additional 24 h in the following media:
(1) medium containing 5% FFA-free BSA as the control
(2) medium containing 5% FFA-free BSA with 50 μM palmitate
(3) medium containing 5% FFA-free BSA with 50 μM stearate

For both experiments, RNA sequencing was conducted on the Illumina NovaSeq 6000 platform to generate 100 bp paired-end reads. Raw reads were processed using Trim Galore software. The cleaned reads were aligned to the human genome GRCh38 (ENSEMBL release 99) using the STAR aligner (v2.7.1a). Transcript quantification was performed using RSEM (v1.3.1).

In the initial experiment, R packages were used for hierarchical clustering (heatmap.2 in version 3.1.1 of the plots package), PCA (prcomp in the Stats R package version 4.1.1), differential expression analysis (Linear Models for Microarray Data, limma version 3.28.14), and the construction of volcano plots. Gene ontology (GO) biological process and KEGG pathway enrichment analyses were performed using iDEP.96 [2] (version 96) installed in RStudio (version 1.2.5). In the subsequent experiment, the same analytical processes were applied, but iDEP 2.01 (version 2.01) was utilized for the GO and KEGG pathway enrichment analyses.

For both experiments, DEGs were identified using a FDR cutoff of 0.05 and a minimum fold change of 1.25. Pathway analysis was conducted using GAGE, with gene sets from the GO biological process database. The gene set size was set at a minimum of 5 and a maximum of 2000, and the pathway significance cutoff (FDR) was set at 0.2.
The sequencing data have been deposited in the Gene Expression Omnibus under accession numbers GSE248408.

Preparation of mouse xenograft models utilizing human ovarian cancer cell lines
The cancer cell lines used for xenografts included OVCAR5, OVCAR8, and SKOV3, along with OVCAR5 and OVCAR8 cells modified for shRNA-mediated SCD knockdown (OVCAR5-shSCD-1, OVCAR5-shCtr, OVCAR8-shSCD-1, and OVCAR8-shCtr). Control and knockdown cells were injected into opposite flanks of the same mouse in the shRNA experiments.
The CAY10566 treatment solution, prepared in 0.5 mg/mL methylcellulose, was administered at a dosage of 2.5 mg/kg every 12 h via oral gavage, after the tumors became palpable.
Tumor volume calculation was performed using the formula (length × width^2)/2. Animals were euthanized by CO_2 inhalation before the tumors exceeded 2 cm in any dimension or before the onset of ulcerations, in line with ethical considerations for animal welfare.

Mouse xenograft models harboring PDXs
After collecting surgical specimens from patients at Kyoto University Hospital, primary xenograft tumors were generated by subcutaneously transplanting tumor sections into NOD SCID mice, followed by resection and cryopreservation at −80 °C using Cell Banker1. PDX72 and PDX82 models were further developed in nude and NOG mice,

respectively, with tumors being dissected and subdivided for implantation into mice along with Matrigel.
Following a 3–5-week establishment period, mice were allocated to treatment groups, with specific dietary regimens (NFD, S-HFD, and O-HFD) initiated alongside the administration of vehicle or CAY10566 via oral gavage every 12 h. The exclusion criteria at randomization included tumors exceeding 5 mm or under 2 mm in diameter. The study included six mice in the PDX72 group and four in the PDX82 group. The CAY10566 administration protocol involved a 2.5 mg/kg dosage every 12 h, using methylcellulose as the vehicle.

References:

1. Yamanoi K*, et al.* Suppression of ABHD2, identified through a functional genomics screen, causes anoikis resistance, chemoresistance and poor prognosis in ovarian cancer. *Oncotarget* **7**, 47620-47636 (2016).

2. Ge X. iDEP Web Application for RNA-Seq Data Analysis. *Methods Mol Biol* **2284**, 417-443 (2021).

Supplementary Text

Patient of PDX82:

A 38-year-old female patient with a G2P1 obstetric history and no significant prior medical conditions presented to the hospital with complaints of abdominal distension and rapid weight gain. Diagnostic paracentesis confirmed the presence of adenocarcinoma. Further imaging studies identified a lesion occupying the pelvic region, and blood tests indicated an elevated tumor marker, CA125, at a concentration of 8,780 U/mL. Subsequently, an immediate diagnostic laparoscopy was performed owing to ovarian cancer suspicion, during which a tissue sample was collected to establish a patient-derived xenograft model, PDX82.

The pathological diagnosis confirmed a high-grade serous ovarian carcinoma, and a dose-dense chemotherapy regimen consisting of taxol and carboplatin (TC) was initiated. Four months after the initial diagnosis, the patient underwent interval debulking surgery, which included a total hysterectomy, bilateral salpingo-oophorectomy, omentectomy, peritoneal stripping, low anterior resection of the rectum, diaphragmatic stripping, and resection of surface metastases on the liver and spleen. The postoperative pathological evaluation revealed a stage of ypT3CN1, classified as FIGO stage 4B.

Four to nine months after diagnosis, adjuvant chemotherapy employing the TC regimen was administered. During a follow-up examination at 24 months post-diagnosis, an elevated CA125 level of 106 U/mL was noted. Magnetic resonance imaging (MRI) revealed the presence of recurrent tumors in the pelvic and para-aortic lymph node regions. Consequently, four cycles of pegylated liposomal doxorubicin and carboplatin (PLD/CBDCA) were administered, achieving complete remission and indicating that the tumor remained platinum-sensitive. Maintenance therapy with laparib was initiated at 28 months post-diagnosis.

As of 80 months post-diagnosis, the patient remains in a disease-free state. Genetic testing was conducted using the NCC Oncopanel FC v.3.0, which identified a BRCA2 mutation, specifically c4936_4939del(p.Glu1646Glnfs*23).

Patient of PDX72:

A 43-year-old female patient, gravida 3, para 3, presented to the hospital with chief complaints of abdominal distension. Elevated levels of the tumor marker CA125 were observed, registering a value of 84 U/mL. Diagnosis of adenocarcinoma was confirmed via abdominocentesis. Further imaging studies led to the diagnosis of stage Ic3 ovarian cancer.

Following one cycle of TC chemotherapy, primary debulking surgery was performed. The postoperative pathological diagnosis revealed ypT3aNxM0. Adjuvant chemotherapy with TC was continued for 24 months from the time of initial diagnosis.

Forty-eight months after diagnosis, a computed tomography scan indicated recurrence at the liver surface and pelvic wall. Despite administering TC chemotherapy, tumor enlargement was observed, leading to a diagnosis of platinum-resistant, progressive disease. At 57 months post-diagnosis, SDS involving hepatic tumor resection, omentectomy, splenectomy, partial resection of the right costal margin of the sternum, and resection of the parietal diaphragm and periesophageal lymph nodes was executed. This operation constituted a complete surgery. Concurrently, a patient-derived xenograft (PDX72) was established from the excised tumor. Postoperative chemotherapy with PLD/CBDCA was administered over three courses.

At 61 months post-diagnosis, imaging studies showed multiple intra-abdominal recurrences. At 63 months, complete cytoreductive surgery involving peritoneal metastasectomy, port removal, appendectomy, and high anterior resection of the rectum was performed. Postoperative chemotherapy was administered with gemcitabine, but subsequent imaging studies indicated recurrence.

Subsequent lines of chemotherapy, including CPT-11, vincristine and prednisone (VP), NDP, paclitaxel and bevacizumab (PTX+Bev), and next-generation taxoids and bevacizumab (NGT+Bev), were continued but were unsuccessful in halting disease progression.

At 95 months post-diagnosis, in search of novel treatment options, a comprehensive cancer gene panel test (FoundationOne® CDx) was performed. Mutations in TP53 (TP53-G187fs*17) and amplification of MDM2 were observed, while microsatellite instability was negative, and BRCA was identified as wild-type. The patient succumbed to the disease 115 months post-initial diagnosis.

Regent or Resource	catalogue numbe	Source			
CAY10566	HY-15823	Cayman Che	Ann Arbor	MA	USA
Methylcellulose	9004-67-5	FUJIFILM Wako		Osaka	Japan
dimethyl sulfoxide (DMSO)	037-24053	FUJIFILM Wako		Osaka	Japan
Palmitate	P0500-10G	Sigma-Aldri	St.Louis	MO	USA
Palmitoleate	76160-1G	Sigma-Aldri	St.Louis	MO	USA
Stearate	S4751-5G	Sigma-Aldri	St.Louis	MO	USA
Oleate	1381-1G	Sigma-Aldri	St.Louis	MO	USA
FA-free bovine serum albumin (FFA-1	A1595	Sigma-Aldri	St.Louis	MO	USA
RPMI-1640 medium	R8758	Sigma-Aldri	St.Louis	MO	USA
fetal bovine serum (FBS)	FB-1061	Biosera		Cholet	France
penicillin-streptomycin	P4458	Sigma-Aldri	St.Louis	MO	USA
DMEM/F12	D9785	Sigma-Aldri	St.Louis	MO	USA
EGF	AF-100-15	Pepro Tech	Rocky Hill	NJ	USA
insulin	I9278	Sigma-Aldri	St.Louis	MO	USA
Hydrocortisone	H0396	Sigma-Aldri	St.Louis	MO	USA
Mycoplasma PCR Detection Kit	G238	Applied Bio	Richmond	BC	Canada
SCD-sh1	TRCN000327814	Sigma-Aldri	St.Louis	MO	USA
SCD-sh2	TRC0000327815	Sigma-Aldri	St.Louis	MO	USA
CHOP-sh1	pDNA(VB90003	Vector Buil	Chicago	IL	USA
CHOP-sh2	pDNA(VB90003	Vector Buil	Chicago	IL	USA
CHOP-shctr	pDNA (VB0100	Vector Buil	Chicago	IL	USA
MTT	M6494	Thermo Fish	Waltham	MA	USA
96-well cell culture plate	655180	Greiner Bio-One		Kremsmunst	Austria
Prism 9.5.1		GraphPad S	Boston	MA	USA
Annexin-V APC apoptosis assay kit	601410	Cayman Che	Ann Arbor	MA	USA
gentleMACS Octo Dissociator with Heater		Miltenyi Biotec		Bergisch Gl	Germany
Tumor Dissociation Kit for humans	130-095-929	Miltenyi Biotec		Bergisch Gl	Germany
MACS® SmartStrainers	130-098-462	Miltenyi Biotec		Bergisch Gl	Germany
MACSQuant flow cytometer		Miltenyi Biotec		Bergisch Gl	Germany
FlowJo software version 10.8.1		FlowJo	Ashland	OR	USA
Mouse Insulin ELISA Kit	AKRIN-011T: 63	Fujifilm Value from Inno		Tokyo	Japan
Triethanolamine-HCl	T0449	Sigma-Aldri	St.Louis	MO	USA
SDS	AM9820	Thermo Fisher Scientific			
TritonX-100	168-11805	Fujifilm Wako Pure Chen		Osaka	Japan
Complete-mini protease inhibitor cock	11836153001	Sigma-Aldri	St.Louis	MO	USA
Bis-Tris gradient polyacrylamide gel	MP42G15	Merck		Darmstadt	Germany
polyvinylidene fluoride membranes	1620177	Bio-Rad Lab	Hercules	CA	USA
Blocking-One	03953-95	Nacalai Tesqe		Kyoto	Japan
gamma H2A.X (phospho S139)	Ab2893	Cell Signali	Nanvers	MA	USA
Cleaved Caspase-3 (Asp175)	9661	Cell Signali	Nanvers	MA	USA
WesternBright ECL kit	K-12045	Advansta	San Jose	CA	USA
ChemiDoc XRS+ system		Bio-Rad Lab	Hercules	CA	USA
Image Lab 2.0 software	SCE_014210	Bio-Rad Lab	Hercules	CA	USA
biotinylated goat anti-rabbit secondary	ab64256	Nichirei		Tokyo	Japan
BZ-9000 microscope		KEYENCE		Osaka	Japan
ImageJ software	ver. 1.54e				
RNeasy® Mini Kit	74104	Quiagen		Venlo	Netherlands
ReverTra Ace qPCR RT Kit	FSQ-101	TOYOBO		Osaka	Japan
PowerUp SYBR Green Master Mix	A25742	Thermo Fish	Waltham	MA	USA
StepOne Plus real-time PCR system		Applied Bio	Foster City	CA	USA

scraper	83.395	Sartedt		Numbrecht	Germany
citric acid monohydrate	035-03495	Fujifilm Wako		Osaka	Japan
trisodium citrate dihydrate	31404-15	Nacalai Tesqe		Kyoto	Japan
Biomasher II		Nippi		Tokyo	Japan
oleate-d9		Cambridge I	Andover	MA	USA
LC-MS/MS system	LC-MS8030+ sys	Shimadzu		Kyoto	Japan
ACQUITY UPLC BEH C18		Waters	Milford	MA	USA
Matrigel Matrix Basement Membrane	354230	Corning		NY	USA
Cell Banker1	CB011	Nippon Zenyaku Kogyo		Fukushima	Japan
Illumina NovaSeq 6000 platform	SCR_016387,	Illumina			
Trim Galore software	SCR_011847				
STAR aligner (v2.7.1a)	SCR_004463				
4-PBA	P21005	Sigma-Aldri	St.Louis	MO	USA

**Diets**	**catalogue numbe**	**Source**			
NFD	F-2	Oriental Yeast		Tokyo	Japan
S-HFD	D12113001	Research Di	New Brunsw	N.J.	USA
O-HFD	HFD32	CLEA Japan		Tokyo	Japan

**Gene**		sequences
GAPDH	forward	5′-ACC ACC CTG TTG CTG TAG CCA A -3′,
	reverse	5′-GTC TCC TCT GAC TTC AAC AGC G -3′
β-actin	forward	5′-CACCTTCCAGCAGATGTCGA-3′,
	reverse	5′-AGCATTTGCGGTGGACGATGG-3′;
SCD	forward	5′-TGCCCACCACAAGTTTTCAG -3′,
	reverse	5′-CATCAGCAAGCCAGGTTTGT-3′;
CHOP	forward	5′-GGTATGAGGACCTGCAAGAGGT -3′,
	reverse	5′-CTTGTGACCTCTGCTGGTTCTG -3′;

**shRNA**	**catalogue numbe**	**sequences**	**Species**	**Source**
SCD-sh1	TRCN000032781	CTACGGC	Human	Sigma-Aldrich
SCD-sh2	TRC0000327815	CGTCCTT	Human	Sigma-Aldrich
SCD-shCtr	SHC202V	CAACAAG	Human	Sigma-Aldrich
CHOP-sh1	VB900039-3848r	TGAACGG	Human	Sigma-Aldrich
CHOP-sh2	VB900039-3854g	ACGGAAA	Human	Sigma-Aldrich
CHOP-shCtr	vb010000-0009m	CCTAAGG	Human	Sigma-Aldrich

**Overexpression Vectors**			
hSCD vector	VB900005-7925zug	Human	VectorBuilder
control GFPexpressing vector	VB010000-9298rtg	Human	VectorBuilder

**Anti-bodies**	**Identifier**	**Source**			**dilution**
Gamma H2A.X (phospho S139)	Ab2893	Abcam	Cambridge	UK	1:1000
Cleaved caspase-3 (Asp175)	9661	Cell Signalir	Danvers	MA, USA	1:1000
SCD1	ab19862	Abcam	Cambridge	UK	1:1000
XBP1	ab37152	Abcam	Cambridge	UK	1:1000
ATF-4	ab184909	Abcam	Cambridge	UK	1:1000
ATF-6	ab37149	Abcam	Cambridge	UK	1:1000
CHOP	15204-1-AP	Proteintech	Chicago	IL, USA	1:1000
α-tubulin	2144	Cell Signalir	Danvers	MA, USA	1:1000

GAPDH	97166	Cell Signalir	Danvers	MA, USA	1:1000
anti-mouse IgG, HRP-linked antibody	7076S	Cell Signalir	Danvers	MA, USA	1:3000
anti-rabbit IgG, HRP-linked antibody	7074	Cell Signalir	Danvers	MA, USA	1:3000
